# Accuracy in Rietveld quantitative phase analysis: a comparative study of strictly monochromatic Mo and Cu radiations

**DOI:** 10.1107/S1600576716003873

**Published:** 2016-04-12

**Authors:** L. León-Reina, M. García-Maté, G. Álvarez-Pinazo, I. Santacruz, O. Vallcorba, A. G. De la Torre, M. A. G. Aranda

**Affiliations:** a Servicios Centrales de Apoyo a la Investigación, Universidad de Málaga, 29071-Málaga, Spain; b Departamento de Química Inorgánica, Cristalografía y Mineralogía, Universidad de Málaga, 29071-Málaga, Spain; c X-ray Data Services S.L., Edificio GREEN RAY, Primera Planta, Avenida Louis Pasteur 47 (Ampliación Campus Teatinos), 29010-Málaga, Spain; d ALBA Synchrotron, Carrer de la Llum 2-26, Barcelona, E-08290 Cerdanyola, Spain

**Keywords:** limit of quantification, spiking method, high-energy laboratory X-ray powder diffraction

## Abstract

This study reports 78 Rietveld quantitative phase analyses using Cu *K*α_1_, Mo *K*α_1_ and synchrotron radiations. Synchrotron powder diffraction has been used to validate the most challenging analyses. From the results for three series with increasing contents of an analyte (an inorganic crystalline phase, an organic crystalline phase and a glass), it is inferred that Rietveld analyses from high-energy Mo *K*α_1_ radiation have slightly better accuracies than those obtained from Cu *K*α_1_ radiation. This behaviour has been established from the results of the calibration graphics obtained through the spiking method and also from Kullback–Leibler distance statistic studies. This outcome is explained, in spite of the lower diffraction power for Mo radiation when compared to Cu radiation, as arising because of the larger volume tested with Mo and also because higher energy allows one to record patterns with fewer systematic errors. The limit of detection (LoD) and limit of quantification (LoQ) have also been established for the studied series. For similar recording times, the LoDs in Cu patterns, ∼0.2 wt%, are slightly lower than those derived from Mo patterns, ∼0.3 wt%. The LoQ for a well crystallized inorganic phase using laboratory powder diffraction was established to be close to 0.10 wt% in stable fits with good precision. However, the accuracy of these analyses was poor with relative errors near to 100%. Only contents higher than 1.0 wt% yielded analyses with relative errors lower than 20%.

## Abstract 2

This work focuses on the comparison of Mo *K*α_1_ and Cu *K*α_1_ radiations for the Rietveld quantitative phase analysis of challenging crystalline mixtures including amorphous content determination. The quantifications have been carried out by using calibration curves with increasing amounts of a given phase. Mo *K*α_1_ patterns were found to yield slightly more accurate analyses than those derived from Cu *K*α_1_ radiation.

## 1. Introduction   

Most industrial materials are multiphase systems and the accurate determination of their phase assemblage is key to understanding their performance. The Rietveld method is nowadays the most employed methodology to achieve quantitative phase analysis (QPA) of crystalline materials in general (Madsen *et al.*, 2001 ▸; Scarlett *et al.*, 2002 ▸) and cements in particular (León-Reina *et al.*, 2009 ▸; Stutzman, 2005 ▸). These inter-laboratory comparisons gave some valuable recommendations for performing accurate Rietveld QPA (RQPA). The factors affecting the accuracy and precision of RQPA results can be gathered into three main groups: (i) instrumental; (ii) sample preparation; and (iii) data analysis protocol(s). The latter is related to the fact that every quantitative X-ray diffraction method requires the scaling of observed diffraction intensities with a suitable analytical standard. The Rietveld method is considered as a standardless methodology as it uses the crystal structure descriptions of each crystalline component to calculate its powder pattern. Consequently, the correct choice of the crystal structure description for each phase in a multiphase system is key (Madsen *et al.*, 2001 ▸; Zevin & Kimmel, 1995 ▸). The influence of the instrument type on the RQPA has been previously evaluated (Madsen *et al.*, 2001 ▸); those authors concluded that both neutron and synchrotron (short-wavelength) powder diffraction yielded the best results, where the obtained values were the closest to the true ones. This was attributed mainly to the higher irradiated volumes, and also to the minimization of the microabsorption effects.

Employing high-energy (short-wavelength) radiation allows us (i) to minimize absorption and microabsorption effects and (ii) to measure a higher number of Bragg peaks (useful mainly for structural studies). In addition, the use of short-wavelength X-rays enables an increase in the specimen irradiated volume. Molybdenum radiation combined with a flat sample in transmission geometry gave an irradiated volume of ∼100 mm^3^, while for copper radiation (flat sample in reflection geometry) the irradiated volume was ∼2 mm^3^ (Cuesta *et al.*, 2015 ▸). In spite of these advantages, the angular resolution may be compromised when using X-rays with short wavelengths owing to the squeezing of the patterns. Consequently, the optics path must include appropriate elements [monochromator(s), slits, collimators *etc.*] to overcome severe peak overlap problems.

It must also be noted that Mo radiation has a major drawback when compared to Cu radiation. The λ^3^ dependence of diffraction intensity favours Cu diffraction by a factor of 10.2. So, a detector receives ∼10 times as many diffracted X-ray photons with Cu as with Mo (this calculation neglects the different fraction of photons lost in the diffractometer optical paths). This drawback could be partially overcome in modern X-ray detectors by increasing the counting time in Mo patterns without reaching prohibitively long values.

In addition, sample preparation for RQPA is very important as the reproducibility of peak intensity measurements is governed by particle statistics (Elton & Salt, 1996 ▸). It is generally accepted that the diffraction intensities have to be collected with an accuracy close to ±1% to obtain patterns suitable for good RQPA results (Dinnebier & Billinge, 2008 ▸). Particle statistics can be improved by (i) using short wavelengths as mentioned above, (ii) spinning the sample continuously during data collection and (iii) milling the sample to reduce the particle size, although this last approach should be executed with caution to avoid peak broadening or amorphization (Buhrke *et al.*, 1998 ▸).

Finally, another important issue in QPA of mixtures is the limit of detection and the limit of quantification. In this context, the limit of detection can be defined as the minimal concentration of analyte that can be detected with acceptable reliability in a sample (Zevin & Kimmel, 1995 ▸). ‘Acceptable reliability’ is a very elusive criterion as it depends upon the type of problem to be tackled. Madsen *et al.* (2001 ▸) assessed the limits of detection at the ∼1 wt% level; limits of quantification were not explicitly mentioned in that paper. Obviously, the limit of detection can be reduced (improved) by increasing the intensity of the X-ray source, for example, using synchrotron radiation.

The aim of this study is to test a simple hypothesis: high-energy Mo radiation, combined with high-resolution laboratory X-ray powder diffraction optics, could yield more accurate RQPA, for challenging samples, than well established Cu radiation procedure(s). In order to do so, three sets of mixtures with increasing amounts of a given phase (spiking method) have been prepared and the corresponding RQPA results have been evaluated with calibration curves (least-squares fit). Since the amorphous content of the single phases was unknown, the independent study of these mixtures does not allow the accuracy of the methodology to be established. The three designed series had increasing complexity. Firstly, a series of crystalline inorganic phase mixtures with increasing amounts of an analyte, from 0.12 to 4.0 wt%, was studied. This series does not represent a great challenge but it should allow us to determine if the Mo *K*α_1_ methodology is as robust as the well established Cu *K*α_1_ methodology. Secondly, a series of crystalline organic phase mixtures with increasing amounts of an organic compound, from 0.12 to 4.0 wt%, was analysed. This series was selected because of the challenge of working with low-absorbing samples that can result in transparency problems in reflection and inhomogeneous loading in narrow capillaries for transmission studies in diffractometers with parallel optics. This type of mixture is obviously of high interest in the pharmaceutical industry. Finally, a third series with variable amorphous ground glass content, from 0 to 32 wt%, was also studied. This is the most challenging work, as for the internal standard approach, the amorphous content is obtained from the small overestimation of the amount of analysed standard with respect to the weighed value. Any error in the procedure propagates to give large deviations in the derived amorphous content. Furthermore, the effect of preferred orientation on RQPA has been considered by including calcite and gypsum in the inorganic mixtures and lactose in the organic ones. Amorphous content determination is important in a number of industries including, but not restricted to, cements, glasses, pharmaceuticals and alloys.

## 2. Materials and methods   

### 2.1. Materials   

Table 1 ▸ shows details of the single phases used in this work: d-(+)-glucose (99%), d-(−)-fructose (99%) and α-lactose monohydrate (≥99%) from Sigma; d-(+)-xylose (>99%) and calcite (>99%) from Sigma–Aldrich; quartz (99.56%) from ABCR; zincite (99.99%) from Aldrich; micronized gypsum marketed by BELITH SPRL (Belgium). Insoluble anhydrite (i-A) was synthesized by heating the micronized gypsum at 973 K for 1 h in a furnace. All the mixtures were prepared by grinding the weighed phases by hand in an agate mortar for 20 min to ensure homogeneity.

#### 2.1.1. Crystalline inorganic mixtures   

A constant matrix of calcite (C), gypsum (Gp) and quartz (Q) was prepared. Then, six samples with known increasing amounts of i-A were produced, labelled as CGpQ_*x*A, where *x* stands for the target insoluble anhydrite content: 0.00, 0.12, 0.25, 0.50, 1.0, 2.0 and 4.0 wt%.

#### 2.1.2. Crystalline organic mixtures   

A constant matrix of glucose (G), fructose (F) and lactose (L) was prepared. Then six samples with known increasing amounts of xylose (X) were produced, labelled as GFL_*x*X, where *x* stands for the target xylose content: 0.00, 0.12, 0.25, 0.50, 1.0, 2.0 and 4.0 wt%.

#### 2.1.3. Variable amorphous content within an inorganic crystalline phase matrix   

A constant matrix of calcite (C) and zincite (Z) was prepared. Then five samples with increasing contents of amorphous ground glass (Gl), obtained by grinding a very thin optical glass plate by hand in an agate mortar for 30 min, were produced. The elemental composition of the ground glass, determined by X-ray fluorescence, was given by García-Maté *et al.* (2014 ▸). The amorphous content was determined by adding ∼20 wt% of quartz (Q) as an internal standard. The mixtures were labelled as CZQ_*x*Gl, where *x* stands for 0, 2, 4, 8, 16 and 32 wt% of ground glass.

### 2.2. Analytical techniques   

#### 2.2.1. Laboratory X-ray powder diffraction   

All single phases and mixtures were studied with both Mo *K*α_1_ (transmission geometry, trm) and Cu *K*α_1_ (reflection geometry, rfl) strictly monochromatic radiations. RQPA was performed for all the patterns to obtain the phase assemblages.

Mo *K*α_1_ powder patterns were collected in transmission geometry (θ/θ), in constant irradiated volume mode, on a D8 ADVANCE (Bruker AXS) diffractometer (188.5 mm radius) equipped with a Johansson Ge(111) primary monochromator, which gives strictly monochromatic Mo radiation (λ = 0.7093 Å). The X-ray tube worked at 50 kV and 50 mA. The optics configuration was a fixed divergence slit (2 mm) and a fixed diffracted beam anti-scatter slit (9 mm). The energy-dispersive linear detector LYNXEYE XE (500 µm), optimized for high-energy radiation, was used with the maximum opening angle. Under these conditions the samples were measured between 3 and 35° (2θ) with a step size of 0.006° and with a total measurement time of 3 h and 5 min.

Cu *K*α_1_ powder patterns, for exactly the same samples, were recorded in reflection geometry (θ/2θ) on an X’Pert MPD PRO (PANalytical BV) diffractometer (240 mm radius) using strictly monochromatic Cu *K*α_1_ radiation (λ = 1.54059 Å) obtained by a Ge(111) primary monochromator. The X-ray tube worked at 45 kV and 40 mA. The optics configuration was a fixed divergence slit (1/2°), a fixed incident beam anti-scatter slit (1°), a fixed diffracted beam anti-scatter slit (1/2°) and an X’Celerator RTMS (real-time multiple strip) detector, working in the scanning mode with the maximum active length. Under these conditions the samples were measured between 6.5 and 81.5° (2θ) with a step size of 0.0167° and with a total measurement time of 2 h and 36 min.

For Mo *K*α_1_ transmission geometry, samples were placed into cylindrical holders between two Kapton foils (Cuesta *et al.*, 2015 ▸). The absorption factor of each sample was experimentally measured by comparing the direct beam with and without the sample (Cuesta *et al.*, 2015 ▸). The amount of sample loaded (which determines the height of the cylinder) in the holders was adjusted to obtain a total absorption μt ≃ 1, which corresponds to an absorption factor of ∼2.7 or 63% of direct beam attenuation. For the organic samples this criterion was not followed as it would lead to very thick specimens. In this case, the maximum holder thickness was used (1.7 mm). For Cu *K*α_1_ reflection geometry, the flat samples were prepared by rear charge of the flat sample holder in order to minimize preferred orientation. In both diffractometers, all the samples were rotated at 10 r min^−1^ during data collection.

The lowest analyte content samples, CGpQ_0.12A and GFL_0.12X, were measured three times using both radiations, Mo *K*α_1_ and Cu *K*α_1_. Regrinding and reloading of the mixtures in the sample holder were carried out prior to every measurement.

Table 1 ▸ also reports the X-ray linear absorption coefficients for all the phases as microabsorption is always a concern in Rietveld X-ray quantitative phase analyses.

#### 2.2.2. Transmission synchrotron X-ray powder diffraction   

The powder patterns of the lowest analyte content samples, CGpQ_0.12A and GFL_0.12X, were also measured using synchrotron radiation. Synchrotron X-ray powder diffraction (SXRPD) data were collected in Debye–Scherrer (transmission) mode using the diffractometer of the ALBA light source (Fauth *et al.*, 2013 ▸). The wavelength, λ = 0.77439 (2) Å, was selected with a double-crystal Si(111) monochromator and determined using the Si640d NIST standard (*a* = 5.43123 Å). The diffractometer is equipped with a MYTHEN-II detector system. The samples were loaded in glass capillaries of 0.7 mm diameter and rotated during data collection to improve diffracting particle statistics. The data acquisition time was 20 min per pattern to attain a very good signal-to-noise ratio (S/N) over the angular range 1–35° (2θ). Three patterns, taken at different positions along the capillaries, were collected for each sample.

SXRPD data for the amorphous content series, CZQ_*x*Gl, were also measured at ALBA. The experimental setup was the same as described just above but the working wavelength was λ = 0.49591 (2) Å.

#### 2.2.3. Data analysis   

The powder patterns for all the samples were analysed by the Rietveld method as implemented in the *GSAS* software package (Larson & Von Dreele, 2000 ▸) by using a pseudo-Voigt peak shape function (Thompson *et al.*, 1987 ▸) with the asymmetry correction included (Finger *et al.*, 1994 ▸) to allow RQPA. The refined overall parameters were phase scale factors, background coefficients (linear interpolation function), unit-cell parameters, zero-shift error, peak shape parameters and preferred orientation coefficient, when needed. The March–Dollase preferred orientation adjustment algorithm was employed (Dollase, 1986 ▸). The modelling direction must be given as input for the calculations. In this case, the directions for the different phases were taken from previous studies. Alternatively, this direction is extracted from the pattern from the analysis of the differences between observed and calculated intensities for non-overlapped diffraction peaks. The crystal structure descriptions used in this study are reported in Table 1 ▸.

In order to provide a single numerical assessment of the performance of each analysis, a statistic based on the Kullback–Leibler distance (KLD) has been employed (Kullback, 1968 ▸). This approach was previously used to evaluate the accuracy of RQPA applied to standard mixtures (Madsen *et al.*, 2001 ▸; Scarlett *et al.*, 2002 ▸; León-Reina *et al.*, 2009 ▸). Both phase-related KLD distances and absolute values of the Kullback–Leibler distance (AKLD) have been calculated. Accurate analyses are mirrored in low values of AKLD.

#### 2.2.4. Amorphous content determination   

The overall amorphous content was determined from the internal standard methodology approach (De la Torre *et al.*, 2001 ▸; Aranda *et al.*, 2012 ▸). Quartz was used as internal standard. If the original sample contains an amorphous phase, the standard will be overestimated in the RQPA. From the (slight) overestimation of the standard, the amorphous content of the investigated sample is derived (De la Torre *et al.*, 2001 ▸).

#### 2.2.5. Scanning electron microscopy characterization   

All single-phase samples were characterized in terms of particle size by scanning electron microscopy (SEM; JEOL JSM 840, Tokyo, Japan). The powders were gold sputtered prior to SEM observation for better imaging. In addition to the as-received gypsum powder, and for the sake of comparison, a second gypsum powder sample, *viz*. a gypsum single crystal (Málaga, Spain) that had been ground in an agate mortar for 10 min, was also characterized by SEM.

## 3. Results and discussion   

### 3.1. Crystalline single phases   

**Figure 1:**
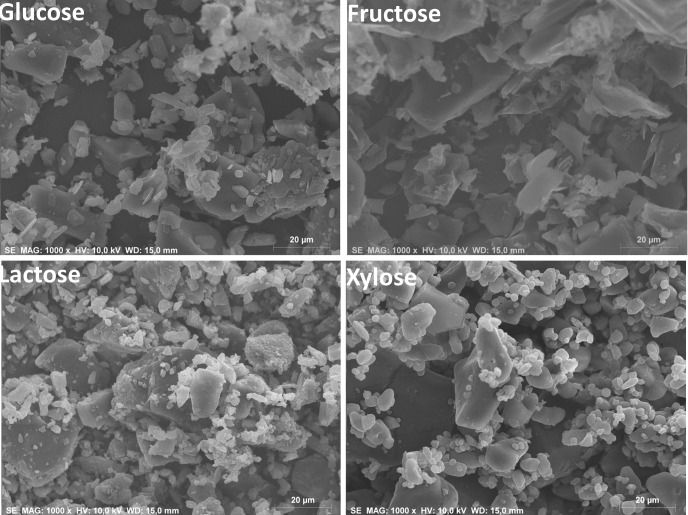
Scanning electron microscopy micrographs for the studied organic phases (×1000).

All the single phases were selected according to several parameters, such as purity, particle size of the powder, preferred orientation and relevance for selected applications. All the phases were previously studied with Mo *K*α_1_ in order to check the suitability of the used crystal structures (see Table 1 ▸). These preliminary studies were of special interest for organic phases as the CIFs obtained from the CSD did not contain the atomic displacement parameters.^1^ For lactose and fructose, the atomic displacement parameters were obtained from the reported data in the original publication and introduced manually in the *GSAS* control file. However, for glucose and xylose phases, these values were not reported in the original publications. Consequently, three groups of isotropic atomic displacement parameters were refined for glucose and xylose: 0.01 Å^2^ as starting value for carbon, hydrogen and oxygen atoms. Table 2 ▸ reports the final atomic displacement parameters for glucose and xylose, as well as *R*_F_ values before and after their optimization, showing the improvements of the fits. The values reported in Table 2 ▸ were obtained from the fits to the Mo *K*α_1_ patterns for the single phases. In the RQPA of the organic mixtures, the atomic displacement parameters were kept fixed to these values. Preferred orientation was modelled by the March–Dollase algorithm along the [001] axis for both glucose and lactose. Table S1, deposited as supporting information, includes final refined profile function parameters and preferred orientation parameters for all the Mo *K*α_1_ refinements for organic single phases. These values were used as starting data to perform the refinements of the organic mixtures. Final Rietveld plots of the four Mo *K*α_1_ patterns for the organic single phases are given as supporting information in Figs. S1–S4. Since microparticle sizes and the distribution of different phases may allow us to explain some sample-related effects, such as preferred orientation, microabsorption and ‘graininess’, all powders were characterized by SEM. Fig. 1 ▸ shows SEM micrographs for all the organic single phases.

**Figure 2:**
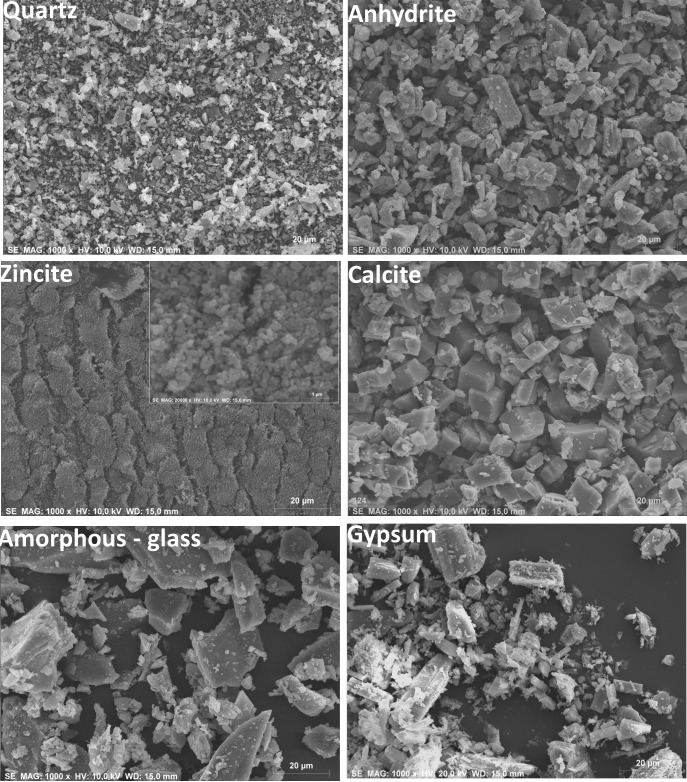
Scanning electron microscopy micrographs for the studied inorganic phases (×1000). The inset of the zincite micrograph shows the powder at higher magnification (×20 000).

The inorganic phases were also analysed by SEM and Mo *K*α_1_. Fig. 2 ▸ reports micrographs for all inorganic phases. Table S2 includes the refined profile function parameters obtained for the inorganic phases analysed with Mo *K*α_1_ radiation. Final Rietveld plots for quartz, calcite, insoluble anhydrite and zincite are given as supporting information in Figs. S5–S8. All inorganic samples were single phases except gypsum and insoluble anhydrite. The gypsum sample used in this work was selected because of its small and homogeneous particle size. Fig. S9 shows SEM micrographs of the gypsum powder used in this study (Fig. S9*a*) and a ground gypsum single crystal (Fig. S9*b*). The latter shows an inhomogeneous particle size distribution and quite large particle sizes, and therefore it was not used. The selected gypsum powder shows a more homogeneous particle size distribution; it contained, as minor phases, 2.25 (4) wt% of soluble anhydrite (s-A) and 1.13 (4) wt% of SrSO_4_, which were considered in the analysis of the mixtures. Table S3 shows the full phase assemblage of the used gypsum powder from the Mo *K*α_1_ RQPA. Table S3 includes refined/used profile function parameters for all the phases. Fig. S9*c* also shows Rietveld plots for gypsum collected with Mo *K*α_1_ radiation.

Both organic and inorganic phases were also measured by using Cu *K*α_1_ radiation in reflection mode. The profile parameters were adjusted and preferred orientation was modelled as in the Mo *K*α_1_ patterns. The transparency effect of light compounds was observed in the Cu *K*α_1_ patterns for organic samples, as expected (Buhrke *et al.*, 1998 ▸). Fig. S10 shows raw Mo and Cu *K*α_1_ patterns for glucose, as an example, to highlight the transparency effect. The peaks in the Cu *K*α_1_ patterns show a strong left-peak asymmetry and some are split, making them relatively difficult to fit.

### 3.2. Limit of detection and quantification   

The limit of detection (LoD) and limit of quantification (LoQ) are two important quantities in any analytical method validation. They have not been widely investigated in powder diffraction but they have been thoroughly used and discussed in the context of analytical measurements of drugs and pharmaceutical compounds [see for instance the review by Shrivastava & Gupta (2011 ▸)]. LoD/LoQ are terms used to describe the smallest concentration of an analyte that can be reliably detected/measured by an analytical procedure. The ‘reliability’ criterion is flexible and may be defined by the regulatory agencies, mainly the case for active pharmaceutical ingredients.

In powder diffraction studies, the LoD for an analyte within a heterogeneous sample can be defined as the minimum amount of the analyte yielding a powder pattern with its strongest (not overlapped) diffraction peak with an S/N larger than 3.0. For techniques such as Rietveld analysis where the full powder pattern is evaluated, this approach is not straightforward. In this context, the LoQ can be defined as the minimum content of an analyte that can be determined with a value at least three times larger than its associated standard deviation and determined to an acceptable reliability level. For RQPA, this type of approach is straightforward although the accuracy for the very low content phases may be quite poor. Finally, and although it may seem counterintuitive, the Rietveld method applied to overlapped powder diffraction patterns may lead to a lower limit of quantification (for the full pattern) than the measured limit of detection, which is (currently) based on single-peak studies.

**Figure 3:**
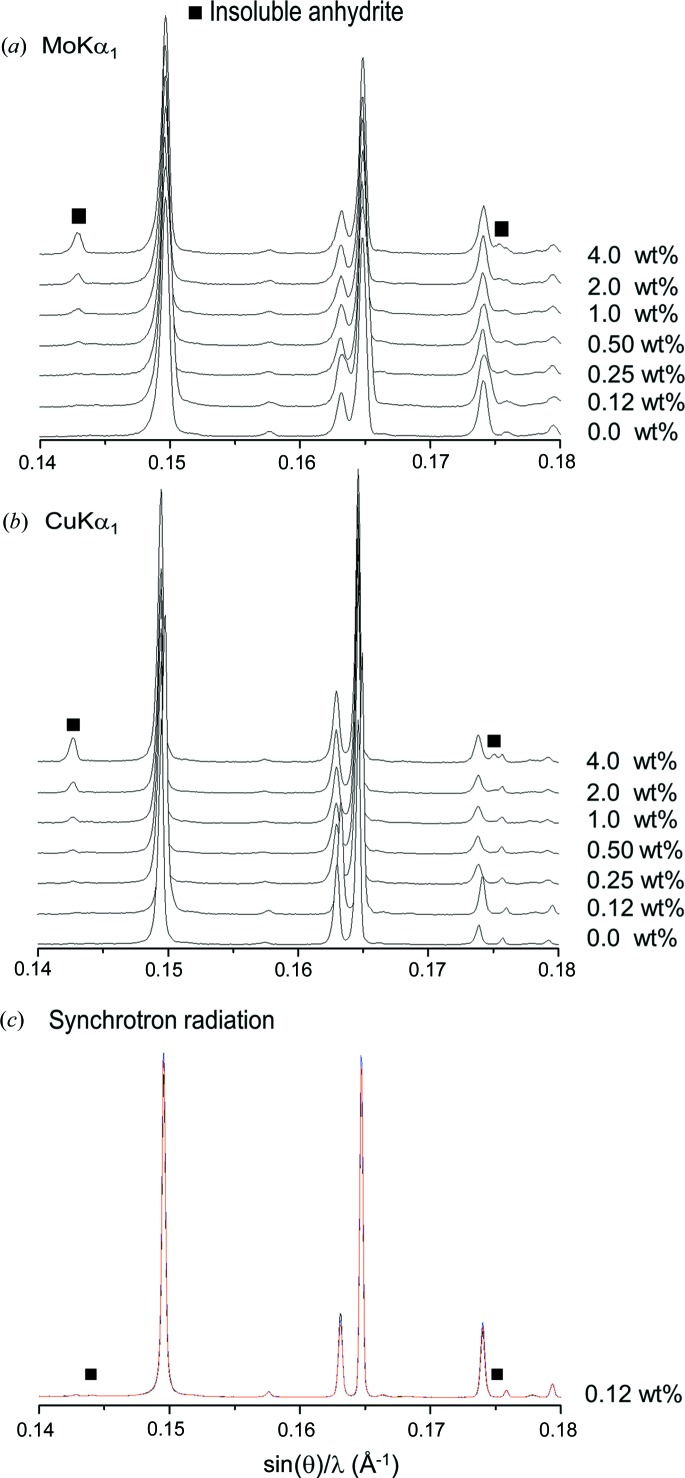
(*a*) Raw Mo *K*α_1_ powder patterns for the inorganic series composed of a constant matrix of calcite, gypsum and quartz and increasing amounts of insoluble anhydrite (peaks highlighted with a solid square). (*b*) Raw Cu *K*α_1_ powder patterns for the same inorganic series. (*c*) Raw SXRPD patterns for CGpQ_0.12A collected at three different positions of the capillary (red, black and blue traces, almost overlapping).

**Figure 4:**
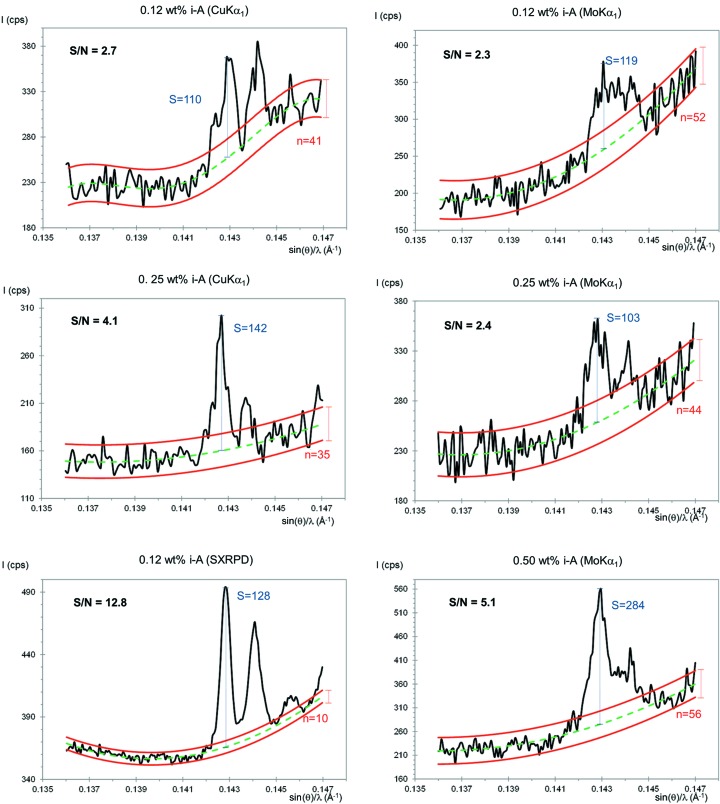
Selected region of the powder patterns showing the main diffraction peak of insoluble anhydrite for the low-content samples used to investigate the limit of detection. Top left: Cu *K*α_1_ pattern for CGpQ_0.12A. Intermediate left: Cu *K*α_1_ pattern for CGpQ_0.25A. Bottom left: SXRPD pattern for CGpQ_0.12A. Top right: Mo *K*α_1_ pattern for CGpQ_0.12A. Intermediate right: Mo *K*α_1_ pattern for CGpQ_0.25A. Bottom right: Mo *K*α_1_ pattern for CGpQ_0.50A. The main peak of anhydrite, sin(θ)/λ = 0.143 Å^−1^, is located at 25.4, 11.6 and 12.7° 2θ for Cu *K*α_1_, Mo *K*α_1_ and synchrotron radiations, respectively. The peak at sin(θ)/λ = 0.1445 Å^−1^ is due to the soluble anhydrite coming from gypsum (constant content in all the samples). The very tiny peak at sin(θ)/λ = 0.1457 Å^−1^, slightly visible only in the SXRPD pattern, arises from SrSO_4_ coming also from gypsum.

Fig. 3 ▸ shows Mo *K*α_1_ and Cu *K*α_1_ raw patterns for the inorganic series with increasing amounts of insoluble anhydrite (highlighted with solid squares). Fig. 4 ▸ shows the strongest diffraction peak for i-A in the mixtures containing 0.12 wt% of anhydrite, CGpQ_0.12A, and 0.25 wt% of anhydrite, CGpQ_0.25A, to evaluate the limits of detection in the conditions reported in §2. For CGpQ_0.12A, both laboratory powder patterns yielded peaks with S/N lower than 3.0 (see top panels in Fig. 4 ▸). For CGpQ_0.25A, its Cu *K*α_1_ pattern yielded a clear peak with S/N = 4.1, so it can be concluded that the LoD for insoluble anhydrite, with this radiation in this mixture, is very close to 0.2 wt%. For Mo *K*α_1_ radiation, the CGpQ_0.25A and CGpQ_0.50A samples yielded patterns with peaks having S/N = 2.4 and 5.1. Therefore, it can be concluded that the LoD for i-A, with this radiation in this mixture, is close to 0.3 wt%.

The LoQ for i-A in this matrix was also studied. We chose to investigate the sample with the lowest anhydrite content to check the influence of using the full powder pattern, although this phase could not be reliably detected by analysing its strongest peak. Three Mo *K*α_1_ patterns and three Cu *K*α_1_ patterns were collected for CGpQ_0.12A. The RQPA results for these analyses, allowing the variation of just the phase scale factor, are reported here. For the three Mo *K*α_1_ patterns, the analysis results for i-A were 0.28 (3), 0.26 (2) and 0.29 (2) wt%. So, the anhydrite content could be quantified, yielding 0.28 (2) wt%, but the accuracy of the obtained value is poor, since the expected value was 0.12 wt%. Similarly, the results for the analyses of the three Cu *K*α_1_ patterns were 0.22 (3), 0.25 (3) and 0.26 (3) wt%, the average value being 0.24 (2) wt%. Full RQPA results are given in Table S4. Therefore, i-A can be quantified in this mixture at the level of 0.12 wt% but with a relative error close to 100%. If the ‘acceptable reliability’ criterion in the analysis were taken into account then the LoQ value would be close to 1.0 wt% as the relative associated error would be lower than 20% (see Table 3 ▸).

CGpQ_0.12A was also studied by SXRPD. Fig. 3 ▸(*c*) shows SXRPD patterns collected in three different positions of the capillary, these patterns being almost identical. The main diffraction peak of anhydrite was clearly observed for this sample (see Fig. 4 ▸ bottom left). The S/N for the strongest diffraction peak of anhydrite was 12.8, and so the limit of detection for i-A, with synchrotron radiation in this matrix, is well below 0.10 wt%. Moreover, Table S5 gives the RQPA results obtained from the three patterns, with very little deviation for all the phases. The average phase assemblage was 34.0 (1) wt% of calcite, 31.4 (4) wt% of gypsum, 33.6 (4) wt% of quartz and 0.20 (1) wt% of insoluble anhydrite. As expected, the accuracy in the SXRPD analysis was better than that attained using laboratory radiation.

To quantify the accuracy of the analysis results shown in Tables S4 and S5, the KLD methodology has been applied. Tables S4 and S5 also report the AKLD values for each analysis as well as the KLD values for the i-A phase in the analysis. The AKLD values for the synchrotron, Mo and Cu radiation analyses are 0.024, 0.031 and 0.057, respectively, these numbers being the average of the results from the three independent analyses for each radiation. The synchrotron analysis is indeed better than the laboratory radiation analyses. Furthermore, the Mo *K*α_1_ radiation analysis is better than the Cu *K*α_1_ one.

**Figure 5:**
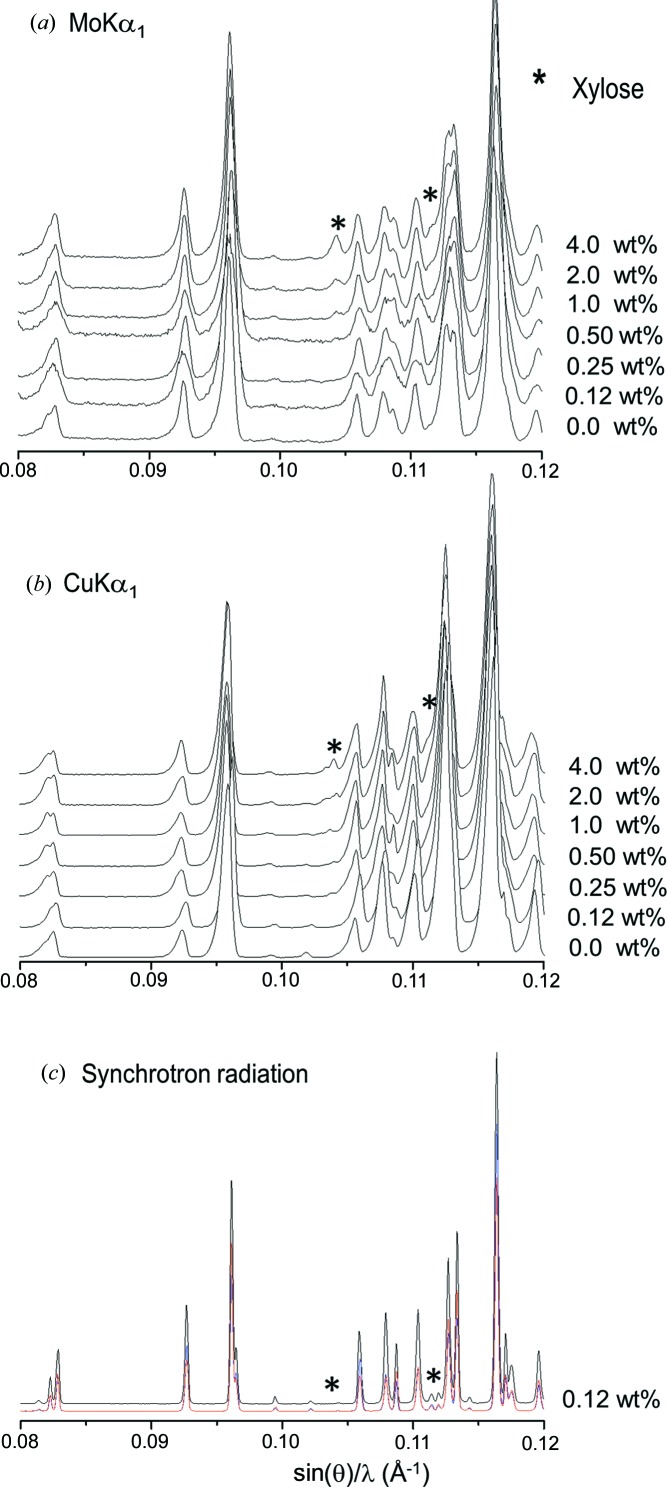
(*a*) Raw Mo *K*α_1_ powder patterns for the organic series composed of a constant matrix of glucose, fructose and lactose and increasing amounts of xylose (peaks highlighted with an asterisk). (*b*) Raw Cu *K*α_1_ powder patterns for the same organic series. (*c*) Raw SXRPD patterns for GFL_0.12X collected at three different positions of the capillary.

Fig. 5 ▸ shows Mo *K*α_1_ and Cu *K*α_1_ raw patterns of the organic mixtures with increasing amounts of analyte (in this case xylose). The strongest powder diffraction peak for xylose was not observed in the GFL_0.12X patterns (both Mo and Cu ones). The corresponding peak was observed in the GFL_0.25X patterns. So, the LoD can be established to be close to 0.25 wt%. The analysis results for xylose in GFL_0.25X (see Table 4 ▸) were 0.33 (4) and 0.57 (9) wt% for the Mo *K*α_1_ and Cu *K*α_1_ patterns, respectively. These values showed that the results for Mo *K*α_1_ were slightly more accurate.

The LoQ for xylose was also studied. Three Mo *K*α_1_ patterns and three Cu *K*α_1_ patterns were collected for GFL_0.12X. The analyses of the three Mo patterns gave 0.17 (5), 0.27 (5) and 0.11 (5) wt% of xylose, and so the average value was 0.18 (8) wt%. Similarly, the results for the analyses of the three Cu patterns were 0.35 (9), 0.28 (10) and 0.40 (8) wt%, the average value being 0.34 (6) wt%. Full RQPA results are included in Table S6. Therefore, the LoD for xylose in this mixture for the two radiations can be established to be close to 0.12 wt%. If one applied an ‘acceptable reliability’ criterion, the LoQ would be much higher, above 1 wt%. Finally, the output using Cu *K*α_1_ radiation is less accurate than that obtained from Mo *K*α_1_ data, although both values were overestimated.

GFL_0.12X was also studied by SXRPD in a rotating capillary in transmission. Fig. 5 ▸(*c*) shows SXRPD patterns for GFL_0.12X collected at three different positions in the same capillary. The powder patterns showed different peak ratios. It is known that filling a capillary with (some) organic compounds is not easy owing to electrostatic charge effects. Furthermore, the phase ratio within the part of the capillary bathed by the X-rays must be the same as that of the sample under study, which cannot be ensured under these circumstances. The differences between the patterns in Fig. 5 ▸(*c*) can be explained by this effect, which results in variable RQPA for the powder patterns of this sample (reported in Table S7). The mean values and the standard deviations of the three analyses, for the three positions, were 35 (4) wt% of glucose, 30 (2) wt% of fructose and 35 (3) wt% of lactose. For samples displaying this behaviour, SXRPD based on glass capillaries is clearly not suitable for obtaining accurate RQPA results. Self-supported sample preparation and other types of holders/capillaries are currently under investigation for pharmaceutical compounds at ALBA, but the results of this ongoing optimization are out of the scope of the present paper.

### 3.3. Increasing inorganic crystalline phase content series   

**Figure 6:**
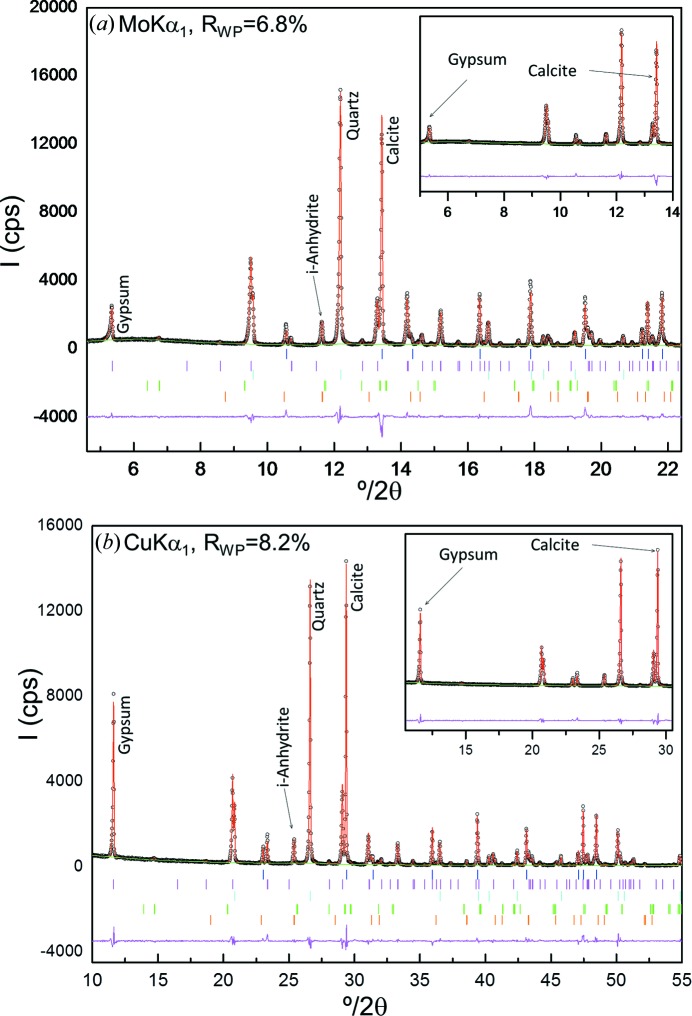
Selected range of the Rietveld plots for CGpQ_4.0A: (*a*) Mo *K*α_1_ and (*b*) Cu *K*α_1_ patterns. The insets highlight the effect of preferred orientation for gypsum and calcite.

Table 3 ▸ reports the RQPA results for the six inorganic mixtures with increasing amounts of i-A measured with Mo *K*α_1_ (in transmission) and Cu *K*α_1_ (in reflection). In general, the values obtained from both radiations are similar. Fig. 6 ▸ displays the Rietveld plots of the mixture with 4 wt% of i-A measured with the two radiations. It must be noted that the gypsum contained soluble anhydrite and SrSO_4_, which were taken into account to calculate the phase assemblage reported in Table 3 ▸. The AKLD values and the KLD values for the i-A phase are also reported in Table 3 ▸. The AKLD values from Mo *K*α_1_ radiation for most of the samples are slightly smaller than the corresponding ones obtained from Cu *K*α_1_ radiation (see Table 3 ▸). Hence, we can conclude that the Mo *K*α_1_ analyses are slightly better than those derived from Cu *K*α_1_.

As discussed above, the investigated samples are exactly the same and their structural descriptions are also identical. Hence, the phase-dependent *R*_F_ agreement factors give an indication of the quality of the data. As an example, Table 5 ▸ reports the *R*_F_ values of all phases for the sample with 4 wt% of insoluble anhydrite. The *R*_F_ values obtained for both patterns are good and quite similar, indicating that both data sets have reproducible peak diffraction intensities.

Furthermore, calcite and gypsum presented preferred orientation, the axis being [104] and [010], respectively. This effect was modelled by using the March–Dollase algorithm (insets in Fig. 6 ▸). Preferred orientation causes the 00*l* reflections for gypsum to have higher intensities in the Cu *K*α_1_ patterns than those calculated from its crystal structure. On the other hand, these reflections in the Mo *K*α_1_ patterns have smaller intensities than those derived from the gypsum structure (see Fig. 6 ▸ top). As a consequence, the refined values for flat samples in reflection and transmission geometries were smaller and larger than 1.0, respectively (Cuesta *et al.*, 2015 ▸). For the mixture with 4 wt% of i-A, as a representative example, the optimized coefficients were 0.815 (2) and 1.200 (4) for gypsum and 0.811 (5) and 1.19 (1) for calcite, in the Cu *K*α_1_ and Mo *K*α_1_ patterns, respectively. Although preferred orientation is present in all patterns, the Cu *K*α_1_ patterns were recorded in reflection geometry (flat samples), while the Mo *K*α_1_ measurements were collected in transmission (also flat samples). This results in opposite diffraction intensity changes and it points towards another (possible) fruitful use: joint refinement of these two types of patterns to counterbalance the effects of preferred orientation in RQPA. Research to fully characterize this is out of the scope of the paper, but we note that it could be helpful in complicated/challenging analyses such as those involving clays/soils.

**Figure 7:**
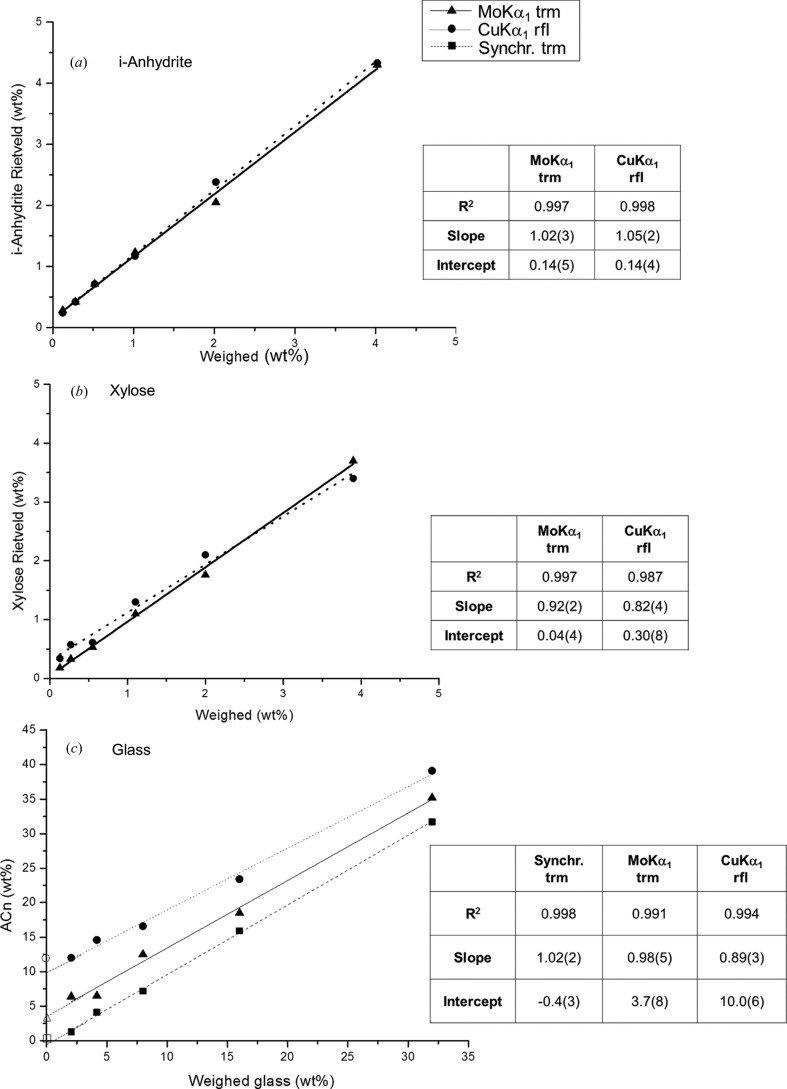
Rietveld quantification results for (*a*) the insoluble anhydrite series (within an inorganic crystalline matrix), (*b*) the xylose series (within an organic crystalline matrix) and (*c*) the ground glass series (within an inorganic crystalline matrix) as a function of the weighed amount of each phase. Open symbols stand for the derived amorphous contents in the mixtures without any added glass. The results of the least-squares fits are also displayed.

Finally, Fig. 7 ▸(*a*) shows the quantified i-A contents, in weight percentage as determined by the Rietveld methodology, as a function of the weighed i-A amount. The inset includes the least-squares fit data. By using the spiking-method approach, the fitted quantitative results are not affected by the possible initial amorphous content present in the employed phases. The two *R* ^2^ values for the fits are very close to 1.00, and the intercept values very close to zero, showing the appropriateness of the Rietveld methodology for quantifying crystalline materials. Furthermore, the slopes of the calibration curves are also 1.00 in both cases, within three times the associated standard deviations. Thus, this study allows us to conclude that RQPA of Mo *K*α_1_ patterns yields results as accurate as, or even slightly better than, those obtained from well established state-of-the-art Cu *K*α_1_ data for crystalline inorganic phases.

### 3.4. Increasing crystalline organic phase content series   

**Figure 8:**
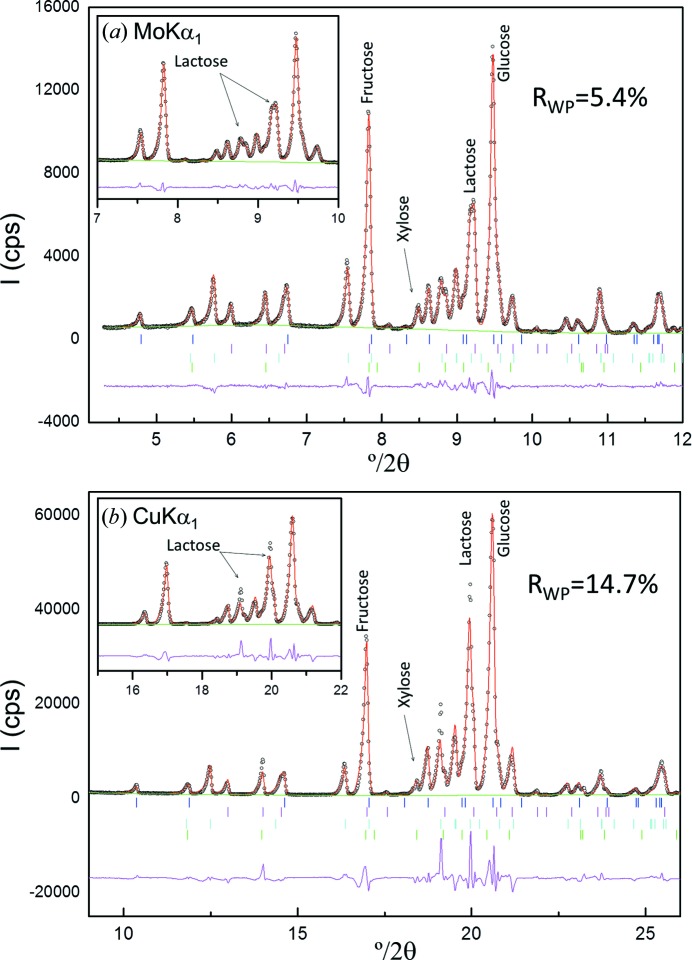
Selected range of the Rietveld plots for GFL_4.0X: (*a*) Mo *K*α_1_ and (*b*) Cu *K*α_1_ patterns. The insets highlight the effect of preferred orientation for lactose.

Table 4 ▸ shows RQPA results of the six mixtures prepared with G, F, L and an increasing amount of X measured with Mo *K*α_1_ (in transmission) and Cu *K*α_1_ (in reflection). In general, the values obtained from both radiations are quite similar to the weighed ones. Fig. 8 ▸ displays the Rietveld plots of the mixture with 4 wt% of xylose for the two studied radiations, as representative examples of the series. The AKLD values and the KLD values for the xylose phase are also reported in Table 4 ▸. The AKLD values from Mo *K*α_1_ and Cu *K*α_1_ radiations are similar.

The main problem for RQPA of organic mixtures measured in reflection geometry is related to the low X-ray absorption of the samples and the transparency effects that lead to poor peak shapes and even some split peaks in the powder patterns. This is shown in Fig. 8 ▸, where the fit of the Mo *K*α_1_ pattern is better (flatter difference curve) than that for Cu *K*α_1_ radiation. Furthermore, as the investigated samples are exactly the same and the structural descriptions are also identical, the phase-dependent *R*_F_ agreement factors are a good indicator of the quality of the recorded data. The lower the *R*_F_ factors, the better the recorded powder diffraction intensities. As a representative example, Table 5 ▸ reports the *R*_F_ values for all phases present in the mixture with 4 wt% of xylose. It is clear that the *R*_F_ values for the organic phases in the Mo *K*α_1_ refinement are lower than those from the Cu *K*α_1_ fit.

The preferred orientation of lactose, along the [001] axis, was modelled by using the March–Dollase algorithm (Dollase, 1986 ▸) (see insets in Fig. 8 ▸). The ellipsoidal corrections for the xylose-free mixture, as a representative example, were 0.947 (7) and 1.110 (3) for the Cu *K*α_1_ and Mo *K*α_1_ patterns, respectively.

Fig. 7 ▸(*b*) shows the quantified xylose contents, in weight percentage as determined by the Rietveld methodology, as a function of the weighed xylose amount added to the mixtures. The inset includes the least-squares fit data. The results were plotted to obtain the calibration lines with increasing content of the analyte. Both curves gave *R* ^2^ values close to 1.0, although the best fit (the *R* ^2^ value closer to 1.0, the slope closer to 1.0 and the intercept value close to 0.0) was obtained for Mo *K*α_1_. This result demonstrates the appropriateness and accuracy of the RQPA with Mo *K*α_1_ for quantifying organic crystalline materials in a routine way (with an easy and reproducible sample preparation methodology).

### 3.5. Increasing amorphous content series within an inorganic crystalline phase matrix   

**Figure 9:**
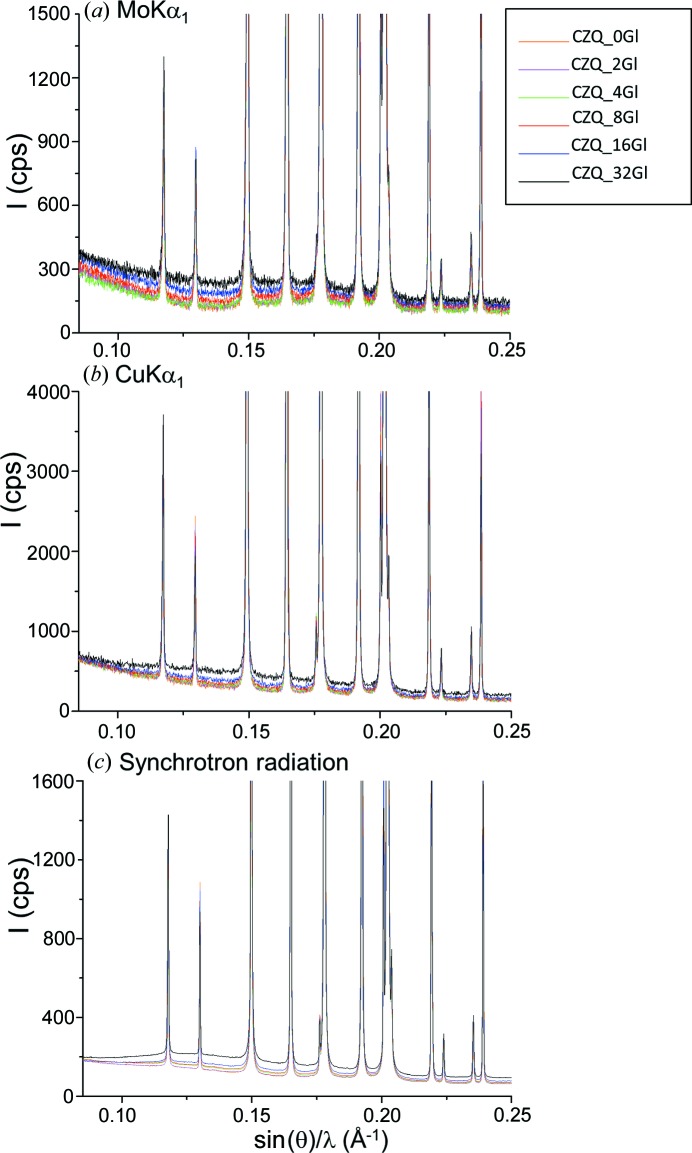
Raw powder patterns for the amorphous-containing series composed of a constant matrix of calcite and zincite and increasing amounts of ground glass. Quartz is added as internal standard. (*a*) Mo *K*α_1_, (*b*) Cu *K*α_1_ and (*c*) SXRPD radiations. The intensities of the patterns have been rescaled to highlight the contributions of the glass to the backgrounds.

Fig. 9 ▸ shows Mo *K*α_1_, Cu *K*α_1_ and SXRPD raw patterns for the mixtures with increasing amounts of glass. We highlight that the increase in the background due to the glass is very modest, even for ∼32 wt% of glass. Table 6 ▸ shows the RQPA of these mixtures, prepared with C, Z and an increasing amount of Gl, measured with synchrotron (in transmission), Mo *K*α_1_ (in transmission) and Cu *K*α_1_ (in reflection) radiations. Weighed amounts are also given for the sake of comparison. The glass-free sample may contain amorphous phase(s) from the employed phases: calcite, zincite and quartz (as an internal standard). Hence, we have used the SXRPD data to calculate a correction factor for quartz to yield zero amorphous content for the glass-free sample. The correction factor for quartz, 1.053, has been derived to give an amorphous fraction for the free-glass sample analysis of 0.4 (1) and an intercept of the calibrating curve of −0.4 (3) (see Fig. 7 ▸ *c*). This factor is applied to the weighed content of quartz prior to the calculation of the amorphous content. The linear fit to the obtained amorphous content values using SXRPD was very good, *R* ^2^ = 0.998, with the slope being 1.00 within the errors (see Fig. 7 ▸ *c*), as expected from an accurate analysis.

**Figure 10:**
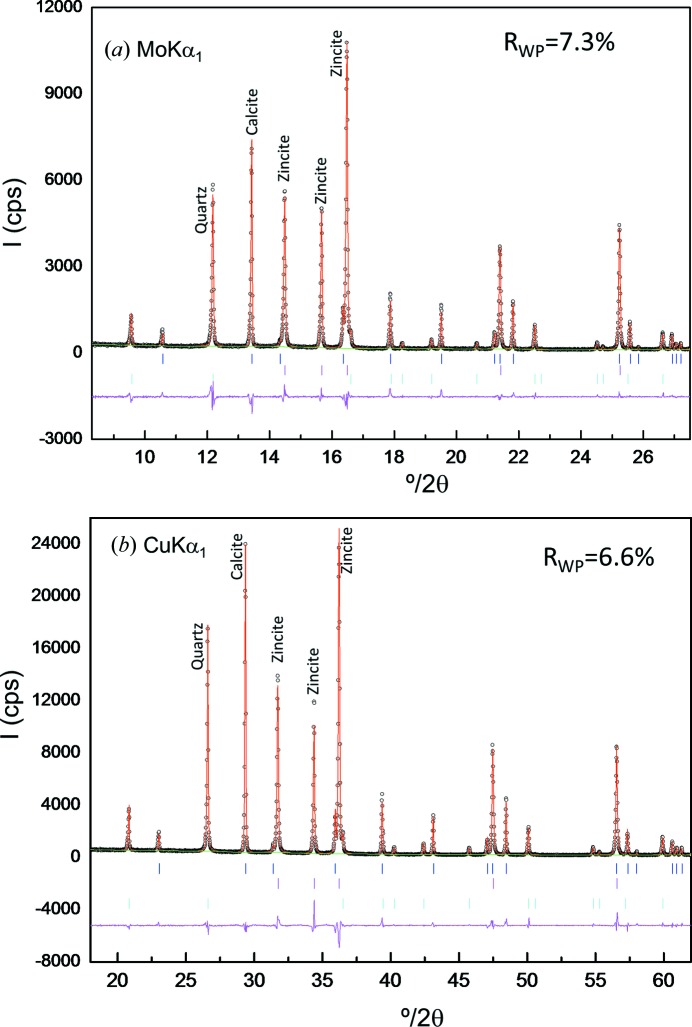
Selected range of the Rietveld plots for CZQ_32Gl: (*a*) Mo *K*α_1_ and (*b*) Cu *K*α_1_ patterns. We highlight that the contribution of 32 wt% of glass to the pattern backgrounds is hardly detectable.

Fig. 10 ▸ displays the Rietveld plots for CZQ_32Gl measured with the two studied laboratory radiations, as an example of this series. As in the inorganic mixtures, calcite presented a preferred orientation along the [104] axis. This effect was also modelled by using the March–Dollase algorithm. For this mixture, the optimized March–Dollase coefficients were 0.858 (6) and 1.21 (2) for calcite in the Cu *K*α_1_ and Mo *K*α_1_ patterns, respectively. Fig. 7 ▸(*c*) shows the quantified amorphous contents, in weight percentage, as a function of the amount of added ground glass, measured with Mo *K*α_1_ and Cu *K*α_1_ radiations. The inset includes least-squares fit data, and open symbols indicate the derived amorphous content obtained with the internal standard method in the mixture without any added glass, CZQ_0Gl. All values reported in Table 6 ▸ and represented in Fig. 7 ▸(*c*) were derived using the correction factor for quartz to ensure zero amorphous content in the glass-free sample. Both *R* ^2^ values are quite close to 1.00, showing the consistency of the internal standard methodology. However, the slope value for the results derived from the Mo *K*α_1_ patterns was 0.98 (5), which was closer to 1.0 than the value obtained from the analyses of the Cu *K*α_1_ patterns, 0.89 (3). Furthermore, the intercept for the Mo *K*α_1_ graph was 3.7 (8) wt%, while the intercept for the Cu *K*α_1_ graph was 10.0 (6) wt%. Moreover, the calculated amorphous values for the glass-free sample were 3.5 and 12.0 wt% for Mo and Cu radiations, respectively. Note that the glass-free values from Mo-based analyses match well (3.7 and 3.5 wt%) and they are relatively close to zero. Meanwhile, there is a larger discrepancy for the similar Cu-based analyses (10.0 and 12.0 wt%), being quite far from zero. Hence, we conclude that the amorphous contents derived from Mo *K*α_1_ data are more accurate than those derived from Cu *K*α_1_ data, probably because of the enhanced particle averaging statistics. Moreover, this systematic study has shown (see Table 6 ▸) that it is not possible to reliably quantify amorphous contents below ∼8–10 wt% from Mo *K*α_1_ data and below ∼15 wt% from Cu *K*α_1_. Conversely, SXRPD allows reliable quantification of amorphous contents up to ∼2 wt% for this relatively simple mixture.

Finally, the AKLD values and the KLD values for the amorphous phase are also reported in Table 6 ▸. The AKLD values for the synchrotron, Mo and Cu radiation analyses are 0.009, 0.057 and 0.169, respectively, these numbers being the average of the results from the six analyses for each radiation. The synchrotron analysis is indeed much better than the laboratory radiation analyses. Furthermore, the Mo *K*α_1_ radiation analyses are also better than the Cu *K*α_1_ ones.

## 4. Conclusions   

(i) We have thoroughly studied the limit of detection for a well crystallized inorganic phase in an inorganic compound matrix. We have determined the following LoDs for insoluble anhydrite: ∼0.2 wt%, ∼0.3 wt% and lower than 0.1 wt% for Cu *K*α_1_, Mo *K*α_1_ and synchrotron radiations, respectively. We conclude that the LoD is slightly better for Cu *K*α_1_ than for Mo *K*α_1_ because the λ^3^ dependence of diffraction intensity, with similar acquisition times, yielded slightly better S/N in the Cu patterns. Of course, detector efficiencies are also playing a role in the measured signal-to-noise ratios.

(ii) We have also studied the limit of quantification for a well crystallized inorganic phase using laboratory X-ray powder diffraction. This phase could be quantified at the level of 0.12 wt% in stable fits with repeatable outputs and good precision. However, the accuracy of these analyses was quite poor with relative errors close to 100%. Only contents higher than 1.0 wt% yielded analyses with relative errors lower than 20%.

(iii) The Rietveld quantitative phase analysis results from high-resolution Mo *K*α_1_ powder diffraction (transmission geometry) and high-resolution Cu *K*α_1_ powder diffraction (reflection geometry) were quite similar for a series of crystalline inorganic phase samples. We inferred from this initial study the validation of the Mo-based analysis procedure, as it yielded very close results to well established high-resolution Cu pattern analyses (see Fig. 7 ▸ *a*). From the comparison of the AKLD values for the two types of analyses, it was deduced that the Mo *K*α_1_ analyses were slightly better than those arising from Cu *K*α_1_.

(iv) The comparison of the results obtained from Mo-based and Cu-based patterns for a series of crystalline organic phase mixtures showed that the Mo *K*α_1_ analyses gave slightly more accurate values. This conclusion was drawn as the calibration curve obtained from Mo patterns with increasing content of xylose gave an *R* ^2^ value closer to 1.0, a slope closer to 1.0 and an intercept value close to 0.0 (see Fig. 7 ▸ *b*). The slightly poorer results from Cu *K*α_1_ analyses are likely to be due to the transparency effects in reflection geometry.

(v) The comparison of the results obtained from Mo *K*α_1_ and Cu *K*α_1_ patterns for a series containing increasing amounts of amorphous glass also indicated that the Mo-based analyses were more accurate. This conclusion was drawn because the obtained calibration curve from Mo data has (i) a slope closer to 1.0, (ii) a smaller value for the amorphous content of the glass-free sample, and (iii) closer agreement between the intercept from the least-squares fit and the calculated amorphous content for the glass-free sample (see Fig. 7 ▸ *c*). The AKLD analysis confirmed this outcome. Furthermore, results from synchrotron powder diffraction have the best accuracy, as shown by the calibration plot and the AKLD analysis.

Finally, we conclude that, for the studied challenging quantification analyses, the results from high-energy Mo *K*α_1_ patterns were slightly more accurate than those obtained from Cu *K*α_1_ patterns. We explain this difference as being the result of the larger amount of tested volume for Mo *K*α_1_ analyses, which leads to better statistics/accuracy in the recorded powder pattern intensities. The absence/minimization of microabsorption in the Mo *K*α_1_ transmission data could very likely be an additional contributing factor to the improved accuracy.

## Supplementary Material

Supporting tables and figures for the article. DOI: 10.1107/S1600576716003873/kc5030sup1.pdf

## Figures and Tables

**Table 1:** Cambridge Structural Database/Inorganic Crystal Structure Database (CSD/ICSD) reference codes for all phases used for Rietveld refinements in this work and linear absorption coefficients for all the used wavelengths

		μ (cm^−1^)		
Phases	CSD/ICSD refcode	Cu *K*α_1_ λ = 1.5406 Å	Mo *K*α_1_ λ = 0.7093 Å	λ = 0.7744/0.4959 Å
Glucose^1^	Glucsa10	12	1	1.3/–
Fructose^2^	Fructo11	12	1	1.4/–
α-Lactose monohydrate^3^	Lactos10	12	1	1.3/–
Xylose^4^	Xylose	12	1	1.2/–
Gypsum^5^	151692	141	16	22/–
Quartz^6^	41414	92	10	11/2.9
s-Anhydrite^7^	16382	219	24	31/–
i-Anhydrite^8^	79527	219	24	31/–
Zincite^9^	65120	285	244	–/89.1
Calcite^10^	80869	194	22	27/7.3
SrSO_4_^11^	22322	299	187	40/–

References: (1) Brown & Levy (1979 ▸); (2) Kanters *et al.* (1977 ▸); (3) Fries *et al.* (1971 ▸); (4) Hordvik *et al.* (1971 ▸); (5) De la Torre *et al.* (2004 ▸); (6) Will *et al.* (1988 ▸); (7) Kirfel & Will (1980 ▸); (8) Bezou *et al.* (1995 ▸); (9) Albertsson *et al.* (1989 ▸); (10) Maslen *et al.* (1995 ▸); (11) Garske & Peacor (1965 ▸); CSD: http://www.ccdc.cam.ac.uk/solutions/csd-system/components/csd/; ICSD: http://www2.fiz-karlsruhe.de/icsd_home.html.

**Table 2:** Refined atomic displacement parameters for glucose (G) and xylose (X) single phases from the Rietveld fit of the Mo *K*α_1_ pattern

	Atomic displacement parameters (Å^2^)			*U*_iso_ = 0.01 Å^2^		*U*_iso_ (refined)	
Phase	C	H	O	*R*_wp_ (%)	*R*_F_ (%)	*R*_wp_ (%)	*R*_F_ (%)
Glucose	0.013 (1)	0.041 (4)	0.0241 (6)	6.0	3.4	5.6	2.8
Xylose	0.023 (1)	0.059 (7)	0.0234 (6)	7.3	5.5	6.9	4.9

**Table 3:** Rietveld quantitative phase analyses for the crystalline inorganic mixtures measured with Cu *K*α_1_ and Mo *K*α_1_ radiations Weighed amounts (%Wt) are shown for the sake of comparison (in bold). The AKLDs for each mixture and the KLD values for i-anhydrite are also included.

	CGpQ_0.0A			CGpQ_0.25A			CGpQ_0.50A			CGpQ_1.0A			CGpQ_2.0A			CGpQ_4.0A		
Phases	%Wt	Mo trm	Cu rfl	%Wt	Mo trm	Cu rfl	%Wt	Mo trm	Cu rfl	%Wt	Mo trm	Cu rfl	%Wt	Mo trm	Cu rfl	%Wt	Mo trm	Cu rfl
C	**32.9**	32.6 (1)	30.4 (2)	**32.8**	32.0 (1)	33.6 (1)	**32.7**	33.2 (1)	32.8 (1)	**32.5**	32.8 (1)	32.6 (2)	**32.2**	31.3 (1)	31.4 (1)	**31.6**	31.2 (1)	31.8 (1)
Gp	**31.7**	31.7 (1)	34.5 (1)	**31.7**	32.5 (1)	31.6 (1)	**31.6**	30.1 (1)	30.7 (1)	**31.5**	30.4 (1)	30.7 (1)	**31.1**	32.1 (1)	32.3 (1)	**30.5**	30.7 (1)	30.5 (1)
Q	**34.2**	34.6 (1)	33.7 (1)	**34.1**	33.9 (1)	33.0 (1)	**34.0**	34.6 (1)	34.2 (1)	**33.8**	34.1 (1)	33.8 (1)	**33.5**	33.5 (1)	32.6 (1)	**32.8**	32.8 (1)	32.0 (1)
s-A	**0.8**	0.66 (3)	0.76 (5)	**0.8**	0.77 (4)	0.78 (5)	**0.8**	0.97 (3)	1.15 (5)	**0.8**	1.03 (4)	1.11 (5)	**0.7**	0.54 (3)	0.58 (5)	**0.7**	0.67 (3)	0.77 (4)
SrSO_4_	**0.4**	0.44 (4)	0.70 (6)	**0.4**	0.44 (4)	0.67 (5)	**0.4**	0.39 (4)	0.56 (5)	**0.4**	0.43 (4)	0.68 (5)	**0.4**	0.48 (4)	0.68 (6)	**0.4**	0.45 (4)	0.63 (5)
i-A	–	–	–	**0.28**	0.42 (3)	0.42 (4)	**0.52**	0.71 (3)	0.71 (4)	**1.02**	1.23 (3)	1.17 (5)	**2.02**	2.05 (4)	2.38 (9)	**4.02**	4.30 (8)	4.33 (9)

AKLD sum		0.0089	0.0605		0.0198	0.0235		0.0295	0.0180		0.0214	0.0152		0.0218	0.0358		0.0095	0.0156
(i-A) KLD					–0.001	–0.001		–0.002	–0.002		–0.002	–0.001		0.000	–0.003		–0.004	–0.003

**Table 4:** RQPA for the crystalline organic mixtures measured with Cu *K*α_1_ and Mo *K*α_1_ radiations Weighed amounts (%Wt) are shown for the sake of comparison (in bold). The AKLDs for each mixture and the KLD values for xylose are also included.

	GFL_0.0X			GFL_0.25X			GFL_0.50X			GFL_1.0X			GFL_2.0X			GFL_4.0X		
Phases	%Wt	Mo trm	Cu rfl	%Wt	Mo trm	Cu rfl	%Wt	Mo trm	Cu rfl	%Wt	Mo trm	Cu rfl	%Wt	Mo trm	Cu rfl	%Wt	Mo trm	Cu rfl
G	**33.4**	33.8 (1)	33.5 (3)	**33.3**	33.6 (1)	33.1 (2)	**33.2**	32.3 (2)	33.5 (2)	**33.0**	34.7 (1)	33.6 (2)	**32.7**	32.2 (1)	31.5 (2)	**32.0**	32.8 (1)	33.6 (2)
F	**33.5**	31.7 (1)	32.7 (3)	**33.4**	32.3 (1)	34.3 (2)	**33.3**	32.1 (2)	33.4 (2)	**33.1**	32.6 (1)	33.7 (2)	**32.8**	31.7 (1)	34.4 (2)	**32.2**	30.7 (1)	32.5 (2)
L	**33.1**	34.5 (1)	33.7 (3)	**33.0**	33.7 (1)	32.0 (2)	**33.0**	35.0 (3)	32.5 (2)	**32.8**	31.6 (2)	31.4 (2)	**32.5**	34.3 (1)	32.0 (2)	**31.8**	32.9 (1)	30.5 (2)
X	–	–	–	**0.27**	0.33 (4)	0.57 (9)	**0.55**	0.53 (8)	0.61 (9)	**1.1**	1.10 (5)	1.3 (1)	**2.0**	1.76 (5)	2.1 (1)	**3.9**	3.70 (5)	3.4 (2)

AKLD sum		0.0362	0.0150		0.0216	0.0231		0.0410	0.0096		0.0338	0.0280		0.0363	0.0339		0.0361	0.0372
(X) KLD		–	–		−0.001	−0.002		0.000	−0.001		0.000	−0.002		0.003	−0.001		0.002	0.005

**Table 5:** *R*_F_ factors of the crystalline phases for the inorganic (CGpQ_4.0A) and the organic (GFL_4.0X) mixtures with ∼4.0 wt% of the minor phase

CGpQ_4.0A†			GFL_4.0X‡		
	Mo *K*α_1_	Cu *K*α_1_		Mo *K*α_1_	Cu *K*α_1_
*R*_F_(C) (%)	3.8	2.8	*R*_F_(G) (%)	1.7	4.5
*R*_F_(Gp) (%)	2.5	2.9	*R*_F_(F) (%)	2.1	4.6
*R*_F_(Q) (%)	1.5	1.3	*R*_F_(L) (%)	1.7	4.2
*R*_F_(s-A) (%)	6.3	6.3	*R*_F_(X) (%)	2.2	5.6
*R*_F_(i-A) (%)	2.6	2.6			
*R*_F_(SrSO_4_) (%)	5.2	3.2			

† The *R*_wp_ values for the Mo *K*α_1_ and Cu *K*α_1_ patterns were 6.8 and 8.2%, respectively.

‡ The *R*_wp_ values for the Mo *K*α_1_ and Cu *K*α_1_ patterns were 5.1 and 13.2%, respectively.

**Table 6:** Rietveld quantitative phase analyses of the CQZ_*x*Gl mixtures, where quartz (Q) is the internal standard to used derive the amorphous content (Am), obtained from SXRPD, Mo *K*α_1_ and Cu *K*α_1_ patterns The AKLDs for each mixture and the KLD values for the amorphous content are also included.

Weighed				SXRPD trm					Mo *K*α_1_ trm					Cu *K*α_1_ rfl				
	C wt%	Z wt%	Gl wt%	C wt%	Z wt%	Am wt%	AKLD sum	Am KLD	C wt%	Z wt%	Am wt%	AKLD sum	Am KLD	C wt%	Z wt%	Am wt%	AKLD sum	Am KLD
CZQ_0Gl	50.01	49.99	**0.00**	49.9 (1)	49.6 (1)	0.4 (1)	0.0050	–	47.5 (1)	49.0 (1)	3.5 (1)	0.0358	–	47.2 (1)	40.8 (1)	12.0 (1)	0.1305	–
CZQ_2Gl	48.98	48.96	**2.05**	49.7 (1)	49.0 (1)	1.3 (1)	0.0169	0.009	45.9 (1)	47.7 (1)	6.4 (1)	0.0679	−0.023	47.4 (1)	40.6 (1)	12.0 (1)	0.1440	−0.036
CZQ_4Gl	47.93	47.91	**4.17**	47.9 (1)	47.6 (1)	4.5 (1)	0.0066	−0.003	46.5 (1)	47.0 (1)	6.5 (1)	0.0422	−0.019	45.8 (1)	39.7 (1)	14.6 (1)	0.1641	−0.052
CZQ_8Gl	46.00	46.00	**7.99**	46.6 (1)	45.9 (1)	7.5 (1)	0.0120	0.005	42.6 (1)	44.8 (1)	12.5 (1)	0.0832	−0.036	45.3 (1)	38.1 (1)	16.6 (1)	0.1522	−0.058
CZQ_16Gl	41.99	41.99	**16.01**	42.0 (1)	41.6 (1)	16.4 (1)	0.0079	−0.004	39.9 (1)	41.7 (1)	18.5 (1)	0.0475	−0.023	40.9 (1)	35.8 (1)	23.4 (1)	0.1388	−0.061
CZQ_32Gl	34.00	34.00	**31.99**	34.0 (1)	33.7 (1)	32.3 (1)	0.0061	−0.003	31.7 (1)	33.1 (1)	35.2 (1)	0.0635	−0.031	32.2 (1)	28.7 (1)	39.1 (1)	0.1403	−0.064

## Footnotes

1 This is important as unaware researchers/analysts downloading CIFs from the CSD lose the atomic displacement parameter values with the current software.
