# Supplementary material for: Accuracy in Rietveld quantitative phase analysis: a comparative study of strictly monochromatic Mo and Cu radiations
Source: J Appl Crystallogr. 2016 Apr 12;49(Pt 3):722–35. doi: 10.1107/S1600576716003873 (PMC4886975; doi:10.1107/S1600576716003873)
Supplement: Supplementary file 1 [file j-49-00722-sup1.pdf]

JOURNAL OF
APPLIED
CRYSTALLOGRAPHY

Volume 49 (2016)

Supporting information for article:

Accuracy in Rietveld quantitative phase analysis: A comparative study of Mo and Cu strictly monochromatic radiations

L. León-Reina, M. García-Maté, G. Álvarez-Pinazo, I. Santacruz, O. Vallcorba, A. G. De la Torre and M. A. G. Aranda

Accuracy in Rietveld quantitative phase analysis. A comparative study of Mo and Cu strictly monochromatic radiations

L. León-Reina[1], M. García-Maté[2,3], G. Álvarez-Pinazo[2,3], I. Santacruz[2], O. Vallcorba[4], A. G. De la Torre[2], M. A. G. Aranda[2,4*]

[1] Servicios Centrales de Apoyo a la Investigación, Universidad de Málaga, 29071-Málaga, Spain.

[2] Departamento de Química Inorgánica, Cristalografía y Mineralogía, Universidad de Málaga, 29071-Málaga, Spain.

[3] X-Ray Data Services S.L., Edificio GREEN RAY, 1ª planta, Avda. Louis Pasteur, 47 (Ampliación Campus Teatinos), 29010-Málaga, Spain.

[4] ALBA synchrotron, Carrer de la Lum, 2-26, E-08290 Cerdanyola, Barcelona, Spain.

This supporting information contains:

Table S1. Refined profile parameters and refined March-Dollase preferred orientation parameter for Mo-Kα_1 patterns of organic phases (Glucose: G; Fructose: F; Lactose: L; Xylose: X).

Table S2. Refined profile parameters and refined March-Dollase preferred orientation parameter for Mo-Kα_1 pattern of inorganic single phases (Calcite: C; Quartz: Q; Insoluble anhydrite: i-A; ZnO).

Table S3. Rietveld quantitative phase analysis, refined profile parameters and refined March-Dollase preferred orientation parameter for Mo-Kα_1 pattern of gypsum sample.

Table S4. Rietveld quantitative phase analysis of CGpQ_0.12A mixture obtained from three independent Mo-Kα_1 and Cu-Kα_1 patterns. Numbers between brackets are the mathematical errors given by Rietveld program calculations.

Table S5. Rietveld quantitative phase analysis of CGpQ_0.12A mixture obtained from SXRPD patterns collected in three different positions in the capillary sample holder. Numbers between brackets are the mathematical errors given by Rietveld calculations. The mean value and the standard deviation of the three analyses are also given.

Table S6. Rietveld quantitative phase analysis of GFL_0.12X mixture obtained from three independent Mo-Kα_1 and Cu-Kα_1 patterns. Numbers between brackets are the mathematical errors given by Rietveld calculations.

Table S7. Rietveld quantitative phase analysis of GFL_0.12X mixture obtained from SXRPD patterns collected in three different positions in the capillary sample holder. Numbers between brackets are the mathematical errors given by Rietveld calculations. The mean value and the standard deviation of the three analyses are also given.

Figure S1. Figure S1. Mo-radiation Rietveld plot for glucose.

Figure S2. Mo-Kα_1 radiation Rietveld plot for fructose.

Figure S3. Mo-Kα_1 radiation Rietveld plot for lactose.

Figure S4. Mo-Kα_1 radiation Rietveld plot for xylose.

Figure S5. Mo-Kα_1 radiation Rietveld plot for quartz.

Figure S6. Mo-Kα_1 radiation Rietveld plot for calcite.

Figure S7. Mo-Kα_1 radiation Rietveld plot for insoluble anhydrite.

Figure S8. Mo-Kα_1 radiation Rietveld plot for zincite.

Figure S9. Scanning electron microscopy micrographs for (a) micronized gypsum, and (b) ground single crystal gypsum. (c) Mo-Kα_1 radiation Rietveld plot for micronized gypsum.

Figure S10. Mo-Kα_1 raw pattern (black) for glucose collected in transmission geometry. Cu-Kα_1 raw pattern (blue) for glucose collected in reflection geometry.

Table S1. Refined profile parameters and refined March-Dollase preferred orientation parameter for Mo-Kα_1 patterns of organic phases (Glucose: G; Fructose: F; Lactose: L; Xylose: X).

Phase	GU /(0.01°)^2	GV /(0.01°)^2	GW /(0.01°)^2	LY /(0.01°)	S/L	H/L	STEC /(0.01°)	PO
**G**	201(13)	-16(3)	2.7(2)	6.7(2)	0.015	0.014	-	1.040(1)*
**F**	159(3)	-	1.93(3)	0.8(2)	0.014	0.015	-	-
**L**	321(17)	-33(4)	3.3(2)	6.0(4)	0.014	0.014	10.6(5)#	1.120(1)*
**X**	377(20)	-36(5)	3.5(2)	3.2(3)	0.015	0.014	-	-

* March-Dollase coefficient along [001] axis. #Anisotropic peak broadening direction for ellipsoidal correction [001].

Table S2. Refined profile parameters and refined March-Dollase preferred orientation parameter for Mo-Kα_1 pattern of inorganic single phases (Calcite: C; Quartz: Q; Insoluble anhydrite: i-A; ZnO).

Phase	GU (0.01°)^2	GV (0.01°)^2	GW (0.01°)^2	LY (0.01°)	S/L	H/L	STEC (0.01°)	PO
**C**	10(5)	-9(2)	2.1(2)	2.7(2)	0.015	0.015	-1.6(3) #	1.050(7)*
**Q**	95(5)	-25(2)	3.2(1)	12.0(2)	0.014	0.014	&	1.120(1)*
**i-A@**	106(2)	-	1.30(4)	2.3(2)	0.015	0.015	8.7(3)ç	1.040(2)$
**ZnO**	32(1)	-	2.03(6)	9.5(2)	0.015	0.015	1.73(6)€	1.040(2)$

*March-Dollase coefficient along [104] axis. $March-Dollase coefficient along [010] axis. #Anisotropic peak broadening direction for ellipsoidal correction [010]. &Anisotropic peak broadening direction for ellipsoidal correction [101] using PTEC (0.01°) parameter: 0.83(7). çAnisotropic peak broadening direction for ellipsoidal correction [100]. €Anisotropic peak broadening direction for ellipsoidal correction [100] jointly with PTEC (0.01°) parameter: 6.4(4). @ It also contains 0.32(3) wt% of SiO_2, 0.86(3) wt% of Cu_5FeS_4 (ICSD code # 1963) and 0.33(5) wt% of $SrSO_4$.

Table S3. Rietveld quantitative phase analysis, refined profile parameters and refined March-Dollase preferred orientation parameter for Mo-Kα_1 pattern of micronized gypsum sample.

Phase	Wt%	GU $(0.01^o)^2$	GV $(0.01^o)^2$	GW $(0.01^o)^2$	LY (0.01^o)	S/L	H/L	STEC (0.01^o)	PO
**Gp**	93.83(2)	129(6)	-13(2)	2.5(1)	6.9(2)	0.014	0.015	-	1.211(2)$^{\$}$
**s-A**	2.25(4)	118(-)	-10(-)	2.2(-)	7.8(-)	0.014	0.015	-	-
**$SrSO_4$**	1.13(4)	133(-)	-27(-)	3.3(-)	15.5(-)	0.014	0.015	-	-
**Q**	0.94(3)	85(-)	-23(-)	3.0(-)	13.0(-)	0.014	0.015	-	-
**C**	0.65(4)	85(-)	-23(-)	3.0(-)	13.0(-)	0.014	0.015	-	-
**i-A**	0.65(3)	85(-)	-23(-)	3.0(-)	13.0(-)	0.014	0.015	-	-
**Na_2SO_4**	0.54(6)	100(-)	-26(-)	3.0(-)	15.0(-)	0.014	0.015	-	-

$^{\$}$March-Dollase coefficient along [010] axis.

Table S4. Rietveld quantitative phase analysis of CGpQ_0.12A mixture obtained from three independent Mo-Kα_1 and Cu-Kα_1 patterns. Numbers between brackets are the mathematical errors given by Rietveld program calculations. Absolute values of the Kullback-Liebler (AKLD) for each mixture and the KLD value for i-anhydrite are also included.

		Mo-LXRPD			Cu-LXRPD		
**Phase**	**Wt%**	**#1**	**#2**	**#3**	**#1**	**#2**	**#3**
**C**	**32.86**	33.6(1)	33.6(1)	33.5(1)	29.9(1)	29.9(1)	30.1(1)
**Gp**	**31.67**	30.4(1)	30.3(1)	30.4(1)	34.1(1)	34.0(1)	33.5(1)
**Q**	**34.17**	34.8(1)	34.9(1)	34.8(1)	34.3(1)	34.4(1)	34.7(1)
**s-A**	**0.79**	0.55(3)	0.58(3)	0.59(2)	0.80(5)	0.77(4)	0.76(5)
**$SrSO_4$**	**0.39**	0.39(4)	0.41(4)	0.41(1)	0.67(5)	0.64(5)	0.59(5)
**i-A**	**0.12**	0.28(3)	0.26(2)	0.29(2)	0.22(3)	0.25(3)	0.26(3)
**AKLDsum**		0.0304	0.0321	0.0291	0.0586	0.0588	0.0547
**(i-A) KLD**		-0.0010	-0.0009	-0.0011	-0.0007	-0.0009	-0.0009

Table S5. Rietveld quantitative phase analysis of CGpQ_0.12A mixture obtained from SXRPD patterns collected in three different positions in the capillary sample holder. Numbers between brackets are the mathematical errors given by Rietveld calculations. The mean value and the standard deviation of the three analyses are also given. Absolute values of the Kullback-Liebler (AKLD) for each mixture and the KLD value for i-anhydrite are also included.

Phase	Wt%	POS1	POS2	POS3	Mean value	Standard deviation
**C**	**32.86**	33.90(5)	34.14(6)	33.97(5)	34.0	0.1
**Gp**	**31.67**	31.24(7)	30.98(7)	31.85(7)	31.4	0.4
**Q**	**34.17**	33.82(5)	33.80(6)	33.14(5)	33.6	0.4
**s-A**	**0.79**	0.64(2)	0.70(2)	0.65(2)	0.7	0.03
**$SrSO_4$**	**0.39**	0.20(1)	0.19(1)	0.20(1)	0.2	0.006
**i-A**	**0.12**	0.21(2)	0.19(2)	0.19(1)	0.2	0.01
**AKLDsum**		0.0210	0.0275	0.0242		
**(i-A) KLD**		0.0006	0.0005	0.0005		

Table S6. Rietveld quantitative phase analysis of GFL_0.12X mixture obtained from three independent Mo-Kα_1 and Cu-Kα_1 patterns. Numbers between brackets are the mathematical errors given by Rietveld calculations. Absolute values of the Kullback-Liebler (AKLD) for each mixture and the KLD value for xylose are also included.

		**Mo-LXRPD**			**Cu-LXRPD**		
**Phase**	**Wt%**	**#1**	**#2**	**#3**	**#1**	**#2**	**#3**
**G**	**33.36**	32.3(2)	33.5(2)	32.4(2)	33.3(3)	31.5(3)	32.8(2)
**F**	**33.46**	32.7(1)	33.1(1)	33.1(1)	34.0(2)	33.9(3)	33.2(2)
**L**	**33.06**	34.8(2)	33.1(2)	34.5(2)	32.4(2)	34.3(3)	33.6(2)
**X**	**0.12**	0.17(5)	0.27(5)	0.11(5)	0.35(9)	0.28(10)	0.40(8)
**AKLDsum**		0.0358	0.0064	0.0276	0.0139	0.0367	0.0151
**(X) KLD**		-0.0004	-0.0010	0.0001	-0.0013	-0.0010	-0.0014

Table S7. Rietveld quantitative phase analysis of GFL_0.12X mixture obtained from SXRPD patterns collected in three different positions in the capillary sample holder. Numbers between brackets are the mathematical errors given by Rietveld calculations. The mean value and the standard deviation of the three analyses are also given.

Phase	POS1	POS2	POS3	Mean value	Standard deviation
**G**	33.0(2)	39.7(2)	32.6(1)	35.1	4.0
**F**	28.4(1)	28.4(1)	32.2(1)	29.7	2.3
**L**	38.6(2)	31.9(2)	35.2(2)	35.2	3.4
**X**	-	-	-	-	-

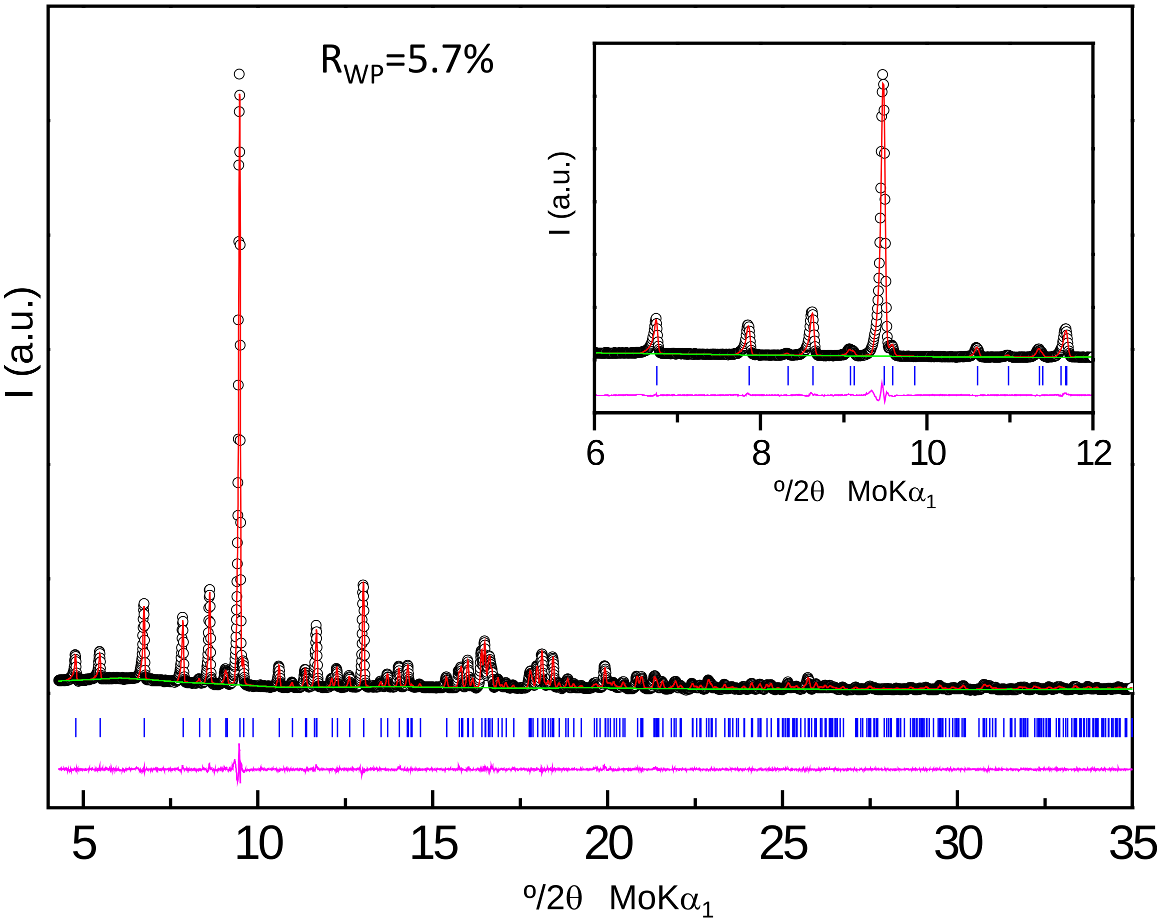

Figure S1. Mo-Kα_1 radiation Rietveld plot for glucose.

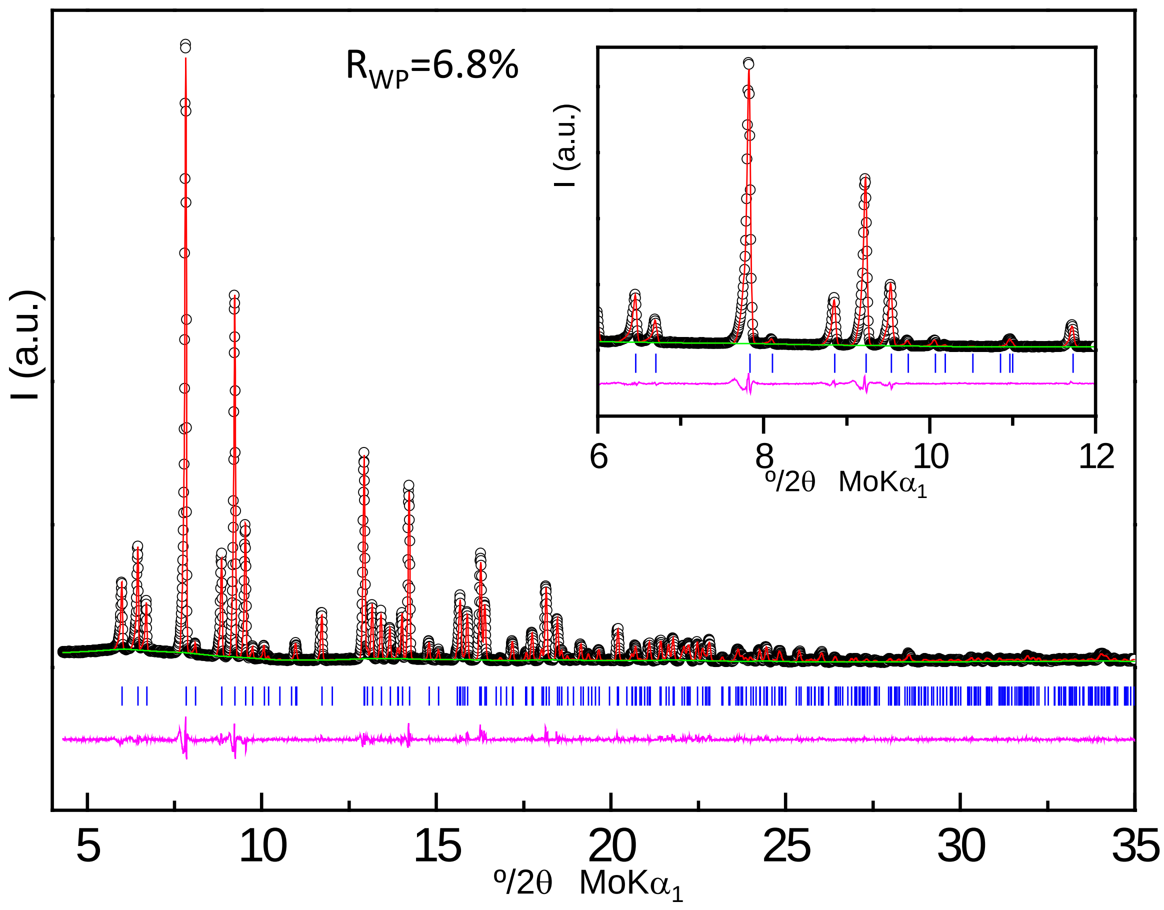

Figure S2. Mo-Kα_1 radiation Rietveld plot for fructose.

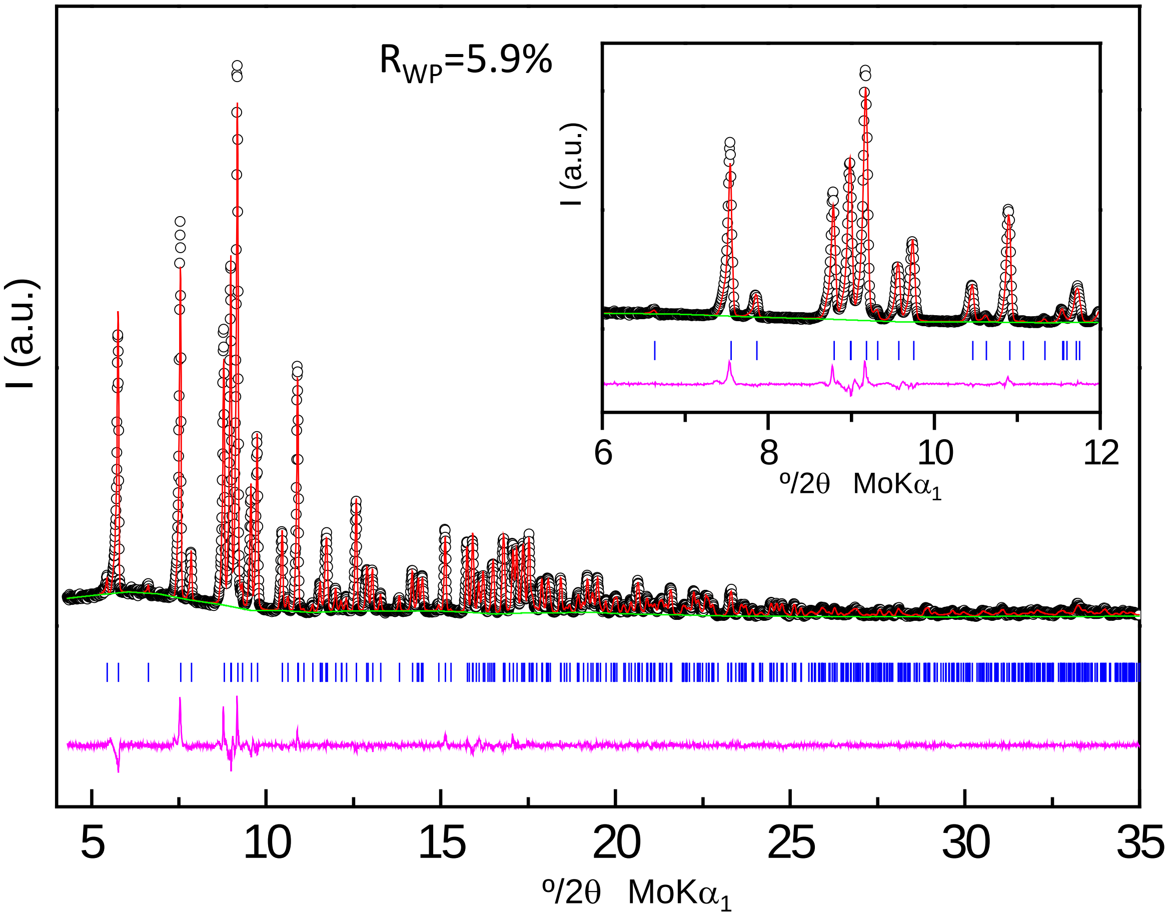

Figure S3. Mo-Kα_1 radiation Rietveld plot for lactose.

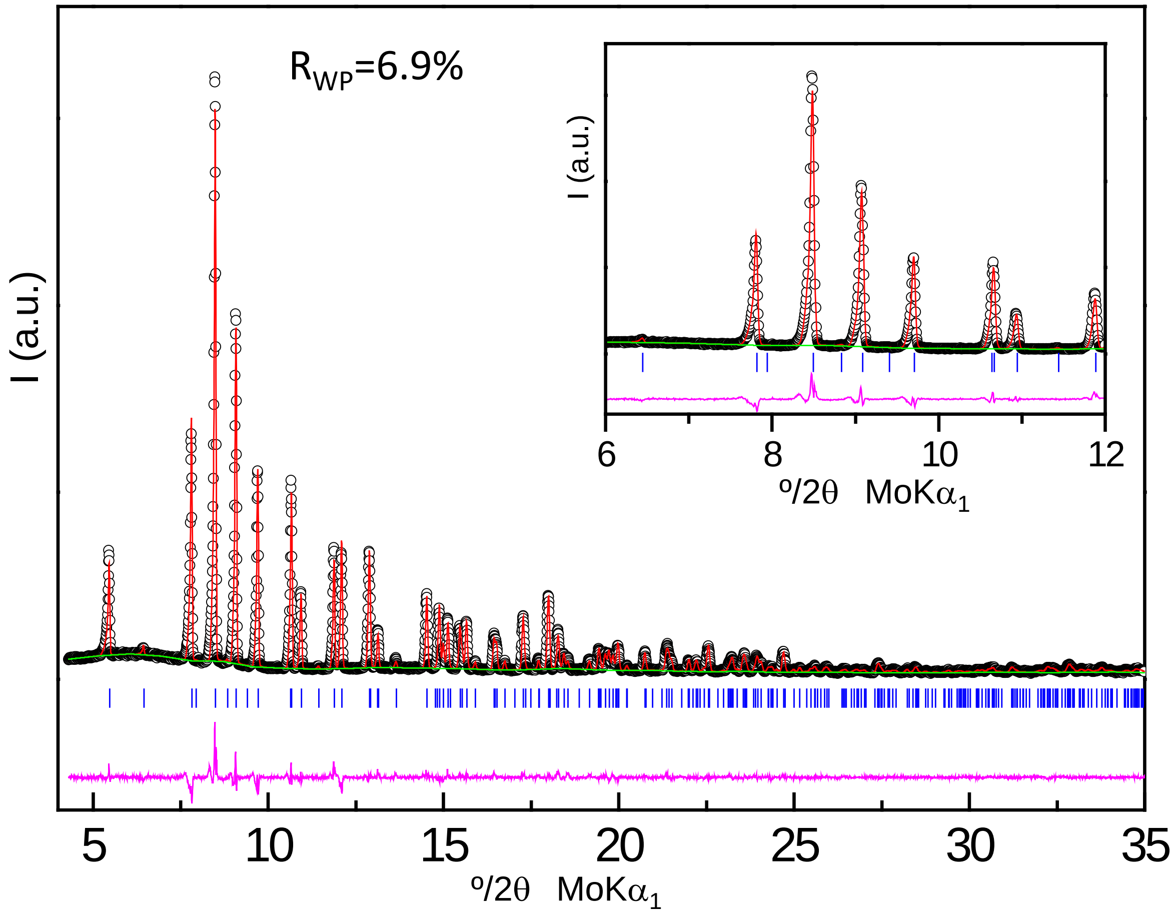

Figure S4. Mo-Kα_1 radiation Rietveld plot for xylose.

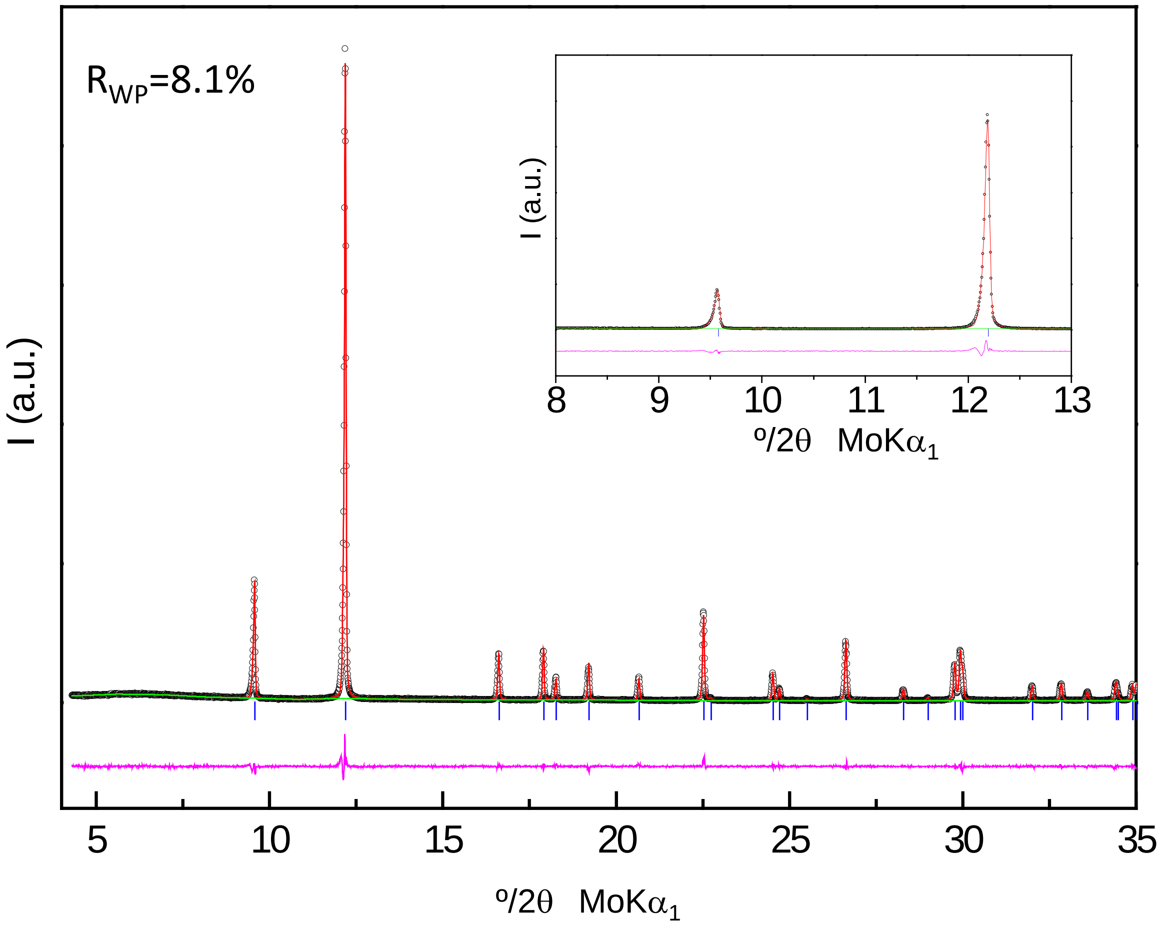

Figure S5. Mo-Kα_1 radiation Rietveld plot for quartz.

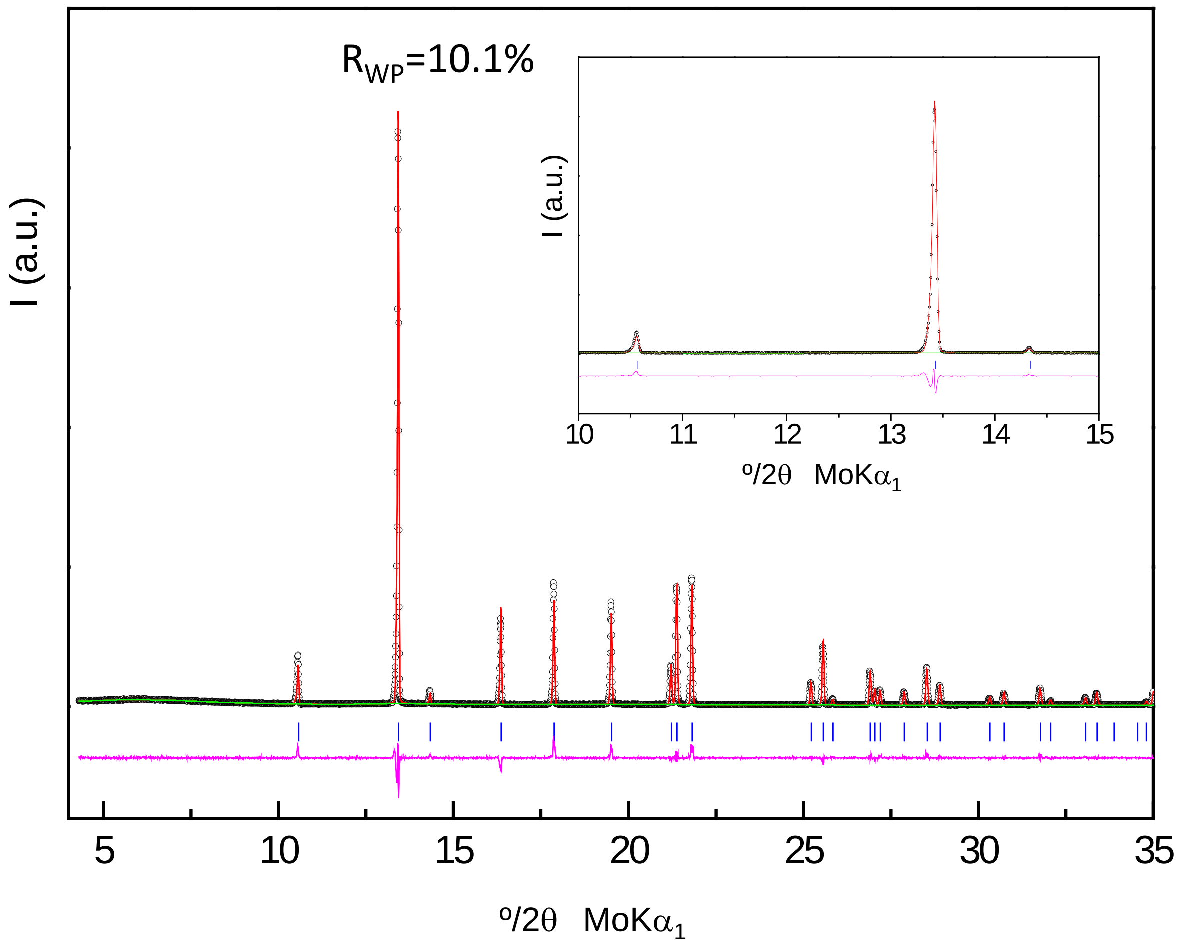

Figure S6. Mo-Kα_1 radiation Rietveld plot for calcite.

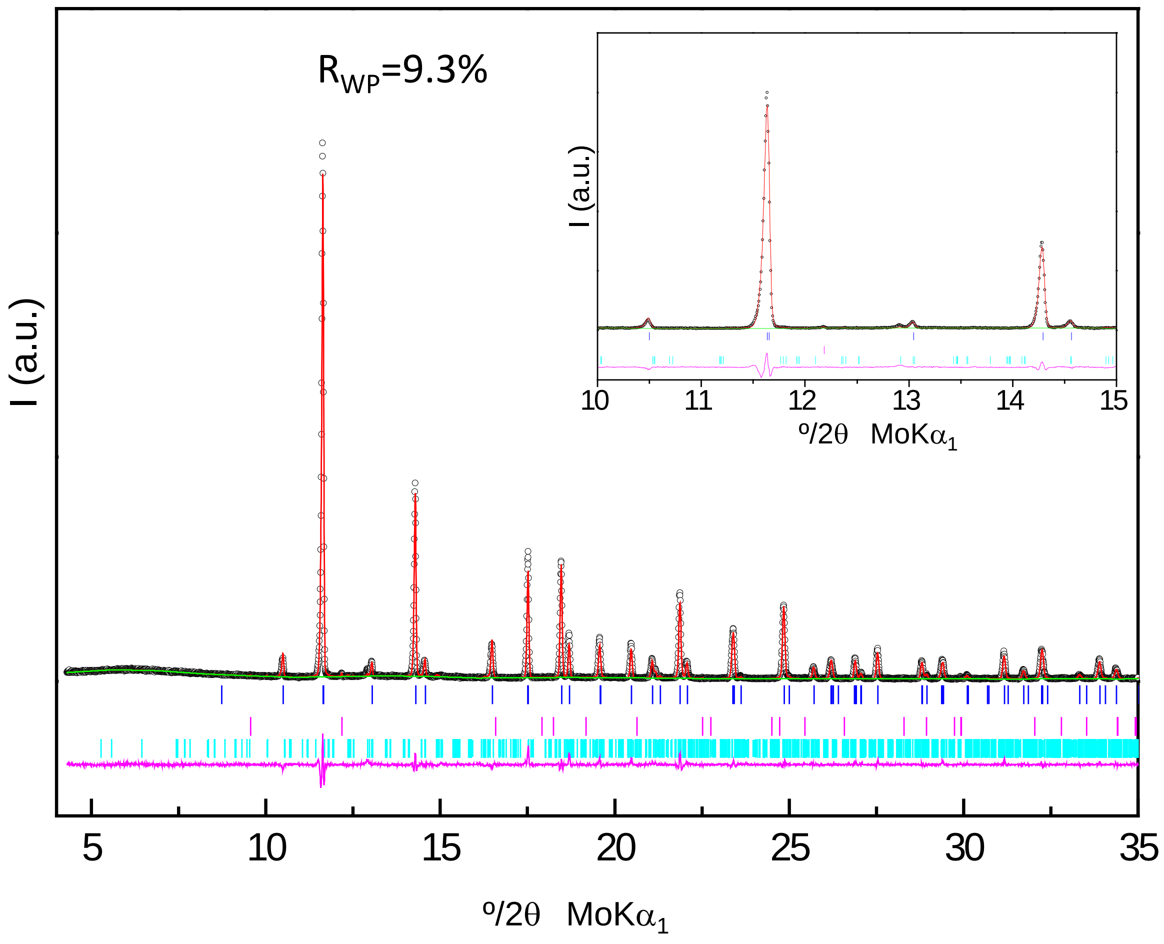

Figure S7. Mo-Kα_1 radiation Rietveld plot for insoluble anhydrite.

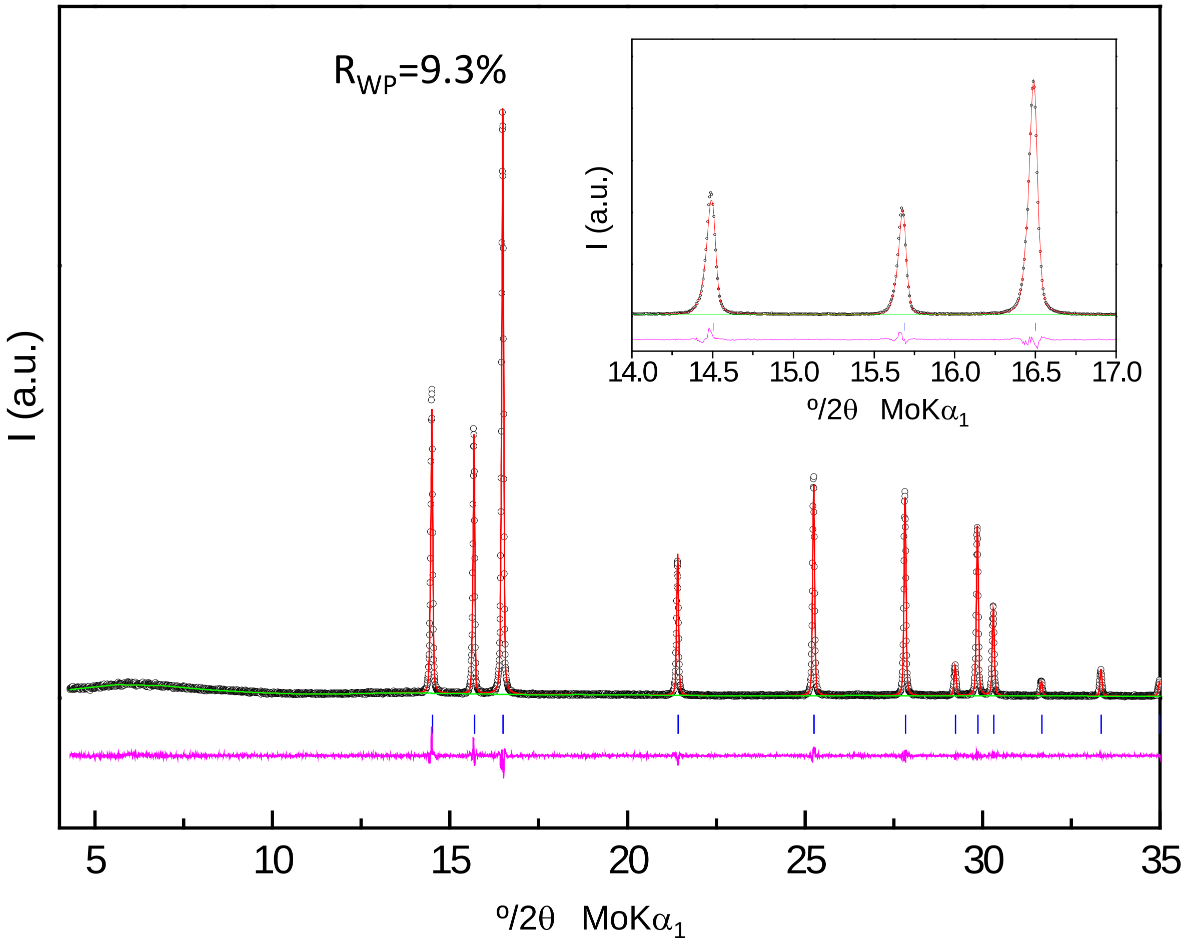

Figure S8. Mo-Kα_1 radiation Rietveld plot for zincite.

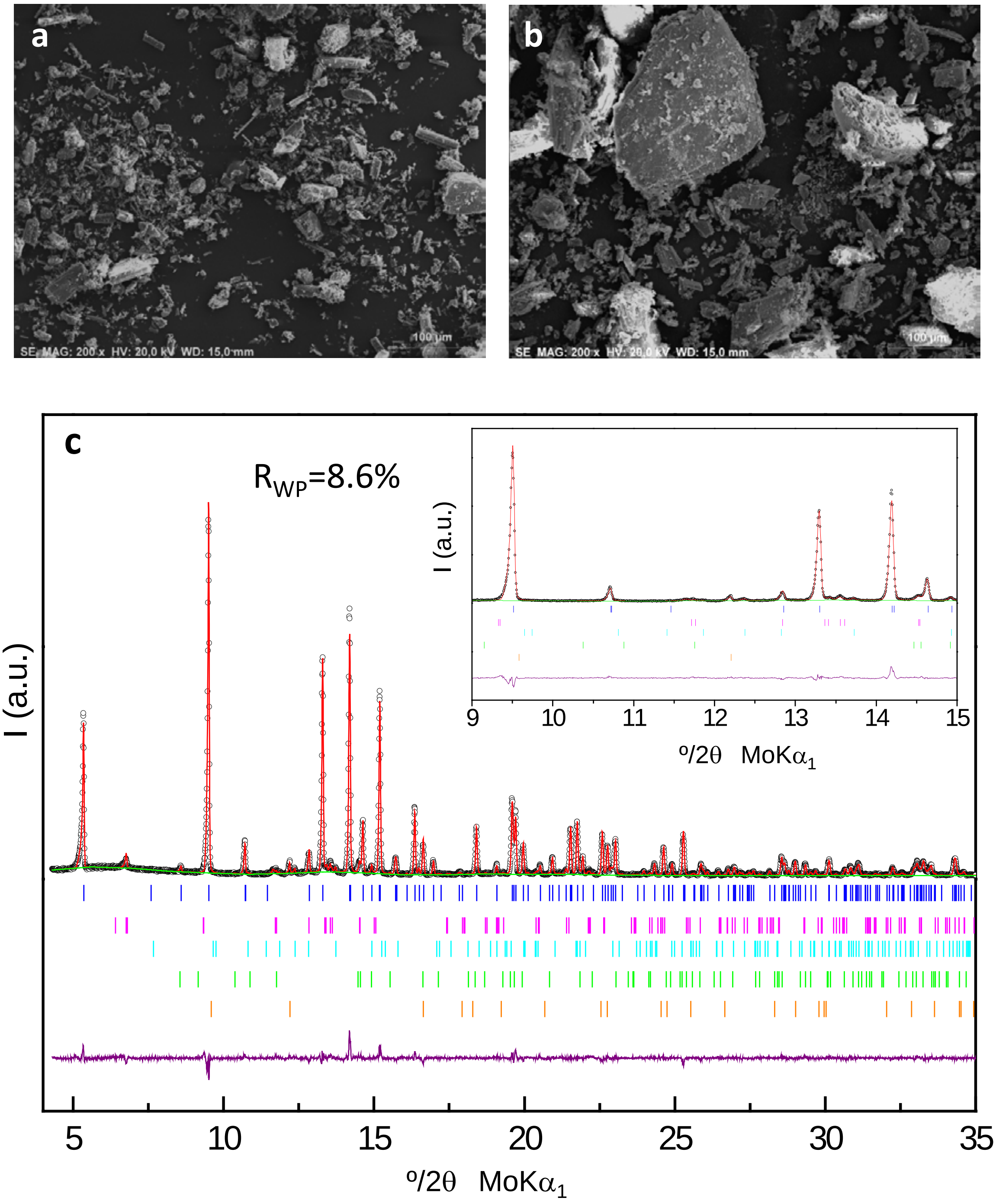

Figure S9. Scanning Electron Microscopy micrographs for (a) micronized gypsum, and (b) ground single crystal gypsum. (c) Mo-Kα_1 radiation Rietveld plot for micronized gypsum.

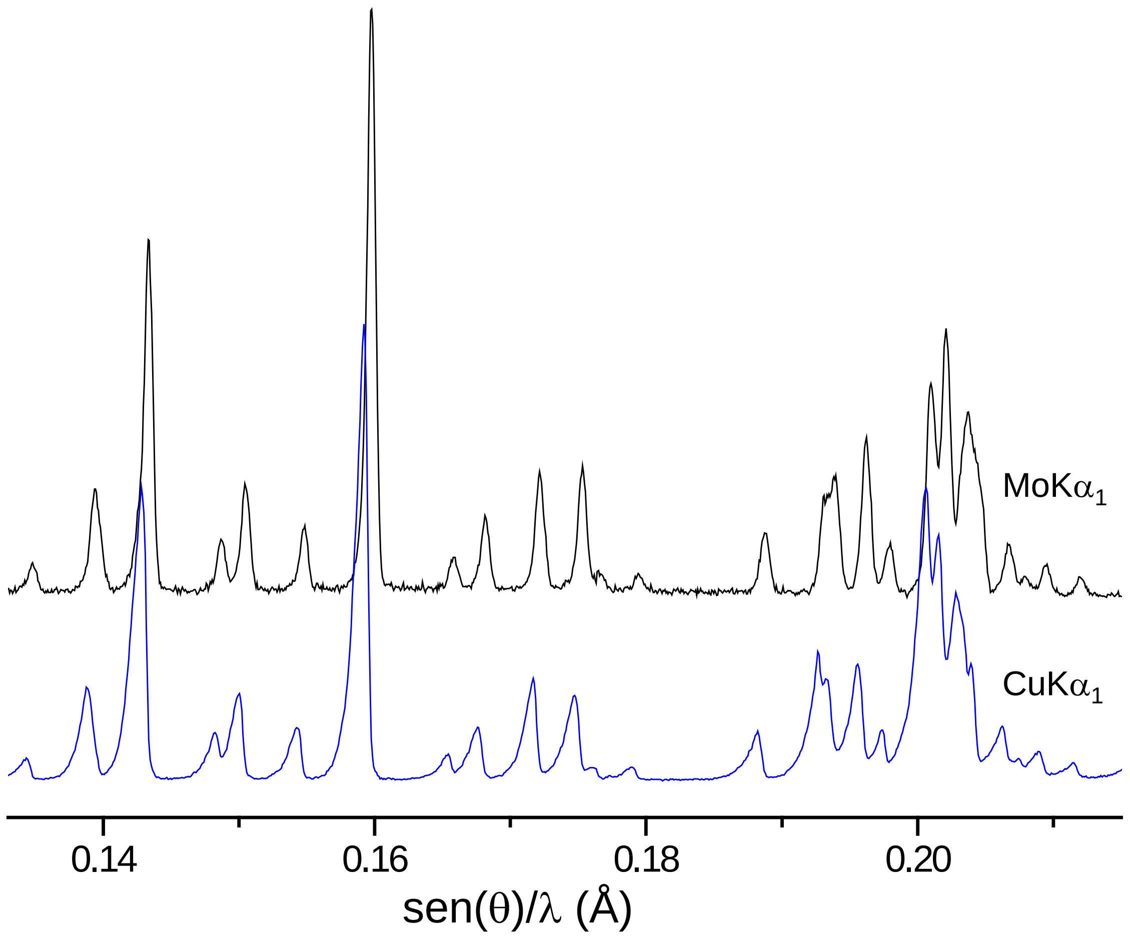

Figure S10. Mo-Kα_1 raw pattern (black) for glucose collected in transmission geometry. Cu-Kα_1 raw pattern (blue) for glucose collected in reflection geometry.
